# Soybean genetic engineering in Brazil and South America - A review

**DOI:** 10.1590/1678-4685-GMB-2025-0007

**Published:** 2026-02-13

**Authors:** Renata Fuganti-Pagliarini, Mayla Daiane Correa Molinari, João Matheus Kafer, Alexandre Lima Nepomuceno, Liliane Marcia Mertz Henning, Sérgio Hermínio Brommonschenkel, Larissa Goulart Zanardo, Maria Helena Bodanese-Zanettini, Elibio Rech

**Affiliations:** 1 Picolla Scientific Consulting, Saskatoon, SK, Canada.; 2 Sempre AgTech, WIN, Chapecó, SC, Brazil.; 3 Embrapa Soja, Londrina, PR, Brazil.; 4 Universidade Federal de Viçosa, Instituto de Biotecnologia Aplicada a Agricultura, Departamento de Fitopatologia Viçosa, MG, Brazil.; 5 Universidade Federal do Rio Grande do Sul, Programa de Pós-Graduação em Genética e Biologia Molecular, Departamento de Genetica, Porto Alegre, RS, Brazil.; 6 Embrapa Recursos Genéticos e Biotecnologia, Instituto Nacional de Ciência e Tecnologia em Biologia Sintética, Brasília, DF, Brazil.

**Keywords:** GMOs, biotechnology, Glycine max, NBTs, biotic and abiotic stresses, herbicide tolerance, drought tolerance, insect resistance

## Abstract

Soybean like other commodities brings important income to producer countries. In Brazil, the picture is not different. Thus, due to its economic significance and considering all field challenges, that impose abiotic and biotic constraints to improve yield, research efforts have focused on solving problems by applying biotechnological tools. In this review, a description of genetic engineering being developed in Brazil for soybean crop is addressed, including research in tolerance to abiotic conditions such as drought; to biotic factors such as insects, nematodes and fungal among other categories; as well as the use of soybean as bio-factories. All commercially available soybean events in Brazil and South American countries, for insect resistance and herbicide tolerance are also discussed. An overview of the Brazilian regulatory framework and South American countries for Biotech products is presented as well as the future perspectives for soybean genetic engineering in Brazil. In addition, a list of papers, from 2008 to the present, showing the state of the art of genetic engineering of soybean and by-products in Brazil is made available. The same search on the literature for South American countries did not return papers published on the genetic engineering of soybean and by-products. Although South American countries did not develop science on GM soybean, a search on GM varieties being sowed indicated that these countries adopted GM technology, due to all the advantages that these lines offer.

## Introduction

Soybeans are among the most important agricultural commodities worldwide, due to their high production volume and central role in food and feed supply chains. For the 2024/25 marketing year, global soybean production is estimated at approximately 420.8 million metric tons, with Brazil contributing about 171.47 million t (CONAB, 2025; USDA, 2025a), followed by the United States with 118.8 Mt and Argentina with 50.9 Mt. However, these yield numbers weren’t always that big. The current world production of soybean is the result of the success, effort, and scientific progress of many researchers, dedicated to basic and applied science, who seek solutions to solve the problems that affect the crop and improve the final productivity, applying alone or in combination classical breeding techniques and/or biotechnology methodologies.

Originally from China, soybean was introduced in Brazil in 1882. Initially, not a prominent crop, interest in soybean foods increased during the 1940s. The 1960s saw a fivefold increase in soybean production in Brazil, from 204,000 tons in 1960 to 1,057.000 tons in 1969. But only in the 70s, soybean production expanded explosively in Brazil, casting the country into world notoriety as a major soybean producer. Between 1970 and 1980, Brazil’s soybean production increased astronomically from 1,2 million to 15,4 million tons. In 1974, our country exceeded China to become the world’s second-largest soybean-producing country, after the USA (Shurtleff and Aoyagi, 2004). This expansion of soybean production was due largely to basic and applied research involving genetic and physiological mechanisms affecting the timing of flowering, plant architecture, productivity, resistance to biotic and abiotic stresses and other adaptive advantages, which allowed the expansion of cultivation areas in Brazil. Thus, much of soybean’s expansion has been due to the quality of Brazil’s national agricultural research (Gavioli, 2013).

The scenario presented above shows that it is not by chance that Brazil became the world’s largest producer with the capacity to continue increasing production in the next decades, even keeping the same planted area (CONAB, 2022). It is important to highlight that soybean promoted other changes in the country, rather than only increasing planted grain area and yield. The explosive growth of soybean production in Brazil (a 30-fold increase across a 30-year period) has profoundly changed Brazilian agriculture. It has boosted farming activities; modernization of the transport system; expansion of the agricultural frontier; professionalization and expansion of international trade; modification and enrichment of the Brazilian diet; acceleration of the country’s urbanization; and population movement from coastal to the interior areas (Gavioli, 2013).

In this work, an overview of former and current soybean research will be addressed. In addition to describing the main soybean research approaches and objectives, to highlight the state-of-the-art genetic engineering of soybean and products resulting from this technology in Brazil, a systematic review to search for academic materials was carried out (Cogo, 2020). The bibliography was selected using the automation software Harzing’s Publish or Perish on the Google Scholar platform (Harzing, 2010). The eligibility criterion consisted of selecting materials published between the years 1980 to 2022 from keywords contextualized to the proposed theme (Cogo, 2020). The search terms used were transgenic OR genetic engineering OR genetically modified AND soybean AND Brazil.

## History of soybean in Brazil

Starting in the early 1970s, Latin America, led by Brazil, began to emerge as a major soybean-producing area. In 1974 Brazil’s production passed that of China and in 1975 Latin America’s total production. The rise of production in Latin America caused America’s share of world production to peak at 76.1% in 1969, falling to a low of 57.5% in 1976. In 1979, Brazil produced 14% of the world’s soybeans (Shurtleff and Aoyagi, 2004), being able to produce nowadays 38% (CONAB, 2022; USDA, 2024).

The story of Brazil’s rise to fame as a soybean producer is an interesting one. Soybeans were being grown commercially on a small scale by the early 1900s, but the crop grew slowly: 8,500 tons a year on average from 1940-49, then 36,000 tons in 1950, 106,000 in 1955, 206,000 in 1960, and 523,000 in 1965. The 1-million-ton mark in Brazilian production was broken in 1969. In 1975, Brazil became the world’s number two producer, with a record harvest of 11.6 million tons (Shurtleff and Aoyagi, 2004). More recently, in the 2024/2025 crop season, Brazil consolidated its position as the world’s largest producer with 171.5 million tons, corresponding to about 41% of global soybean production (CONAB, 2025; USDA, 2025a). This extraordinary success happens due to a long list of reasons, among them the introduction of liming and correction of soil fertility; tax incentives; increased use of vegetable oil vs. animal fats; crop mechanization; the emergence of dynamic and efficient cooperatives; establishment of a well-coordinated network of research; and improvements in roads, ports, and communications (Gavioli 2013; Florentino et al., 2022).

In addition, other scenarios also accelerated the growth of Brazilian soybean production such as the government, which looking for export crops to generate currency offered subsidies and price supports to soybean farmers; Japanese research Institutes offered technical assistance to increase soybean production on marginal frontier land, and Brazil’s oilseed industry began crushing soybeans in the late 1960s. Besides that, in 1973, came the USA soybean export embargo, which artificially raised world prices until it became profitable for even the most inefficient producer to grow soybeans. As an extra benefit, Brazilian soybean shows a large percentage of oil and protein, about 4.5% more oil and 4.5% more protein than US soybeans, being in demand on world markets and attracting a premium price.More recent analyses indicate that soybean meal produced in the United States typically contains around 48% protein on a dry‐matter basis, while Brazilian soybean meal is slightly higher in some cases, although variation depends on genotype, growing conditions, and processing (Oviedo-Rondón et al., 2024, Liu et al., 2024).

Last, but not least important to the establishment of soybean as a major Brazilian crop, was the development of varieties adapted to the short photoperiods of central and northern national areas, allowing the crop to expand, in the 70s, to the “Cerrado” region of Brazil, an area of more than 200 million hectares of undeveloped potential cropland. Research development of these varieties began at the Agronomic Institute (IAC) in Campinas-SP, and the National Center for Soybean Research, by crossing North American cultivars, which had the long juvenile trait. Currently, thousands of soybean genotypes are maintained in the Embrapa Soybean (CNPSo) germplasm bank in Londrina, Paraná State. The entire germplasm collection is maintained at the Embrapa Genetic Resources in Brasília-DF. Most of these accessions are plant introductions from North America which are derived from original plant introductions from China, Japan, and other countries with wide genetic diversification (Gavioli, 2013). 

## Plant Biotechnology - development of GM soybean 

January 18, 1983, is arguably one of the most important dates in the history of plant biotechnology. At the Miami Winter Symposium, three independent groups described *Agrobacterium tumefaciens*-mediated genetic transformation, leading to the production of normal, fertile tobacco transgenic plants. This scientific milestone opened to the scientific community the possibility of applying this technology to other plant species, including soybean, a worldwide commodity, allowing the introduction of agronomically important genes into species that are susceptible to agrobacterial infection (Vasil, 2008). Within the next decades, world scientists worked in all major crop species with genes that conferred resistance to non-selective herbicides, and some pests and pathogens. In the meantime, in response to apprehensions about the safety and environmental impact of biotech crops, elaborate and stringent mandatory regulations were developed in the USA and some other countries to monitor and regulate the cultivation and use of biotech crops and byproducts. 

Plant biotechnology applied to soybean came of age only in 1996, with the planting of nearly five million acres of biotech crops, mostly in the United States (Vasil, 2008). This GM soybean was Monsanto’s Roundup Ready (RR) variety, generated by the introduction of the EPSPS (5-enol-pyruvylshikimate-3-phosphate synthase) gene, which encodes an herbicide-insensitive target-site enzyme. This trait allows soybean plants to survive applications of glyphosate, a non-selective herbicide with a broad spectrum of action in weed control (Shurtleff and Aoyagi, 2020), although resistant or tolerant weed populations have been reported worldwide (Heap, 2025). The same variety of transgenic soybean was first marketed in Argentina in 1997, and in Brazil, after approval by the National Biosafety Technical Commission (CTNBio - in Portuguese Comissão Técnica Nacional de Biossegurança), in 1998. However, in our country due to actions before the Brazilian justice, the decision was put in discussion for seven years. Only in 2005, after the enactment of Law 11,105, the new Brazilian biosafety law, the 1998 decision was confirmed. The RR technology allowed the use of more efficient chemical herbicides with less toxicity. In practice, it also enabled no-tillage in Brazil ensuring better soil water preservation, greater health, better control and weed management, as well as greater carbon sequestration in the soil, helping to reduce greenhouse (GH) gas emissions.

Since 1994 when the first GM product (GMO tomato) was made available to producers, the adoption of GM soybean with different traits spread all around the world, with numbers reaching an area of 90,7 million hectares (Mhas) of GM planted in 2014; 95,5 Mhas in 2018; and 91,9 Mhas in 2019 (ISAAA, 2019; Brookes, 2020). Specifically in Brazil, the area planted with GM soybean varieties was estimated at approximately 45.7 million hectares in 2024, reflecting the very high adoption rate of GE soybean seeds in the country (≈99%) (AgbioInvestor, 2024; USDA, 2025b).

After RR soybean, Intacta Roundup Ready 2 Pro (IPRO or RR2) was launched in 1998, with caterpillar control and glyphosate tolerance. In the 2020/21 crop season, more than 35 Mhas were sown with this technology throughout Latin America. Intacta 2Xtend, launched this 2021/22 season, brought three new genes (*Cry1A.105*, *Cry2Ab2* and *Cry1Ac* genes) to increase soybean resistance to caterpillars, also carrying tolerance to dicamba (3,6-dichloro-2-methoxybenzoic acid) herbicide. After these two pioneer GM soybeans, many others were developed and are available to Brazilian growers today. Currently, 24 GM soybean events are approved for commercialization in Brazil by CTNBio (CTNBio, 2024) and will be detailed later, including herbicide-tolerant cultivars such as Enlist E3 (glyphosate, 2,4-D, and glufosinate) and stacked traits like Conkesta Enlist E3 that combine insect resistance with herbicide tolerance. 

## Biosafety of GMOs in South America - an overview 

After the USA, South America is the region with the greatest expansion of agricultural land cultivating genetically modified crops. In 2023, 97.3 million hectares (Mhas) were seeded with GM crops in South America (AgbioInvestor, 2024). Countries that grow GM crops legally are Argentina, Brazil, Chile, Colombia, Paraguay, and Uruguay. 

In Ecuador and Venezuela, the cultivation of GMOS is banned, however, the importation is allowed. In Peru, though increasingly ignored, cultivation of GM crops is banned but imports of GM soy are allowed (Genetic Literacy Project, 2024). No information regarding the status of GM soybean cultivation in Guyana and Suriname was found (ISAAA, 2024). 

In 2023, Argentina grew 23.1 Mhas of cotton, maize, soybean, and wheat GM crops (AgbioInvestor, 2024). This country was one of the first countries to have a regulatory framework for genetically modified (GM) crops for agricultural use. Initially, the National Advisory Commission on Agricultural Biotechnology (CONABIA- Comisión Nacional Asesora de Biotecnología Agropecuaria) oversaw the entire regulatory and evaluation process, but as activities increased and technologies evolved, the current Innovation and Biotechnology Coordination began to monitor and evaluate the applications submitted to develop activities with GMOs, together with CONABIA (CONABIA, 2024). Briefly, the regulatory process in Argentina is established in Resolution 763/2011 issued by the Ministry of Agriculture. It establishes a procedure divided into three steps: (1) an environmental assessment performed by CONABIA, (2) a food and feed safety evaluation performed by the National Agri-Food Health and Quality Service (SENASA - Servicio Nacional de Sanidad y Calidad Agroalimentaria), and (3) an evaluation of its impact on the agricultural market. Once each step is completed, a Decision Document is drafted, which must be favorable for the event to be approved (Lewi and Vicién, 2020). 

Chile does not cultivate GM crops commercially; but the country allows the propagation of GM seeds for export markets. Typically, GM seed stock is imported from the USA, reproduced and multiplied, and then exported to the USA and Canada (AgbioInvestor, 2024). 

Soybean is the only GM crop cultivated in Bolivia. 1.5 million hectares were cultivated in 2023 (AgbioInvestor, 2024). In Bolivia, although, primarily, the government positions against the technology, the National Biosafety Committee worked on establishing the regulatory framework for the cultivation and commercialization of GM soybean, to fulfill farmers’ requests, who used to grow GM crops by smuggling varieties of transgenic seeds, including soybeans, from neighboring countries. Currently, the use of GMOs is restricted in Bolivia, where GM corn and soybeans are allowed to be planted and it’s regulated by Supreme Decree n° 2452 from 2015 (Bolivia, 2015).

Colombia cultivated 152 thousand hectares of GM cotton and maize in 2023 (AgbioInvestor, 2024). Regarding biosafety, this country was one of the leading countries in formulating the Cartagena Protocol. This country regulates all GMO-related activities. Three different Technical Committees issue their recommendations to the National Competent Authority regarding the approval of applications after a GMO biosafety risk assessment is done. 

The technical councils are: Agricultural Biosafety National Technical Council - Agricultural NTC, Livestock-Animal Biosafety National Technical Council - Livestock NTC, and a Specialized Office on Foods and Alcoholic Beverages - SEABA - of the National Institute on Food and Drug Inspection - INVIMA (in Spanish - Instituto Nacional de Vigilancia de Medicamentos y Alimentos). The general procedure for obtaining the approval of a GMO request in Colombia follows a few steps. Initially, a request is filed and registered. It’s revised regarding compliance with requirements and complete documentation. After that, if applicable, the Ministry of Social Protection makes an assessment. An evaluation of risks derived from the proposed activity is conducted and a report to NTC is filled up. The evaluation is on an individual basis - case by case by NTC, which determines if experimental or field biosafety studies are required and/or requesting additional information to the applicant. Finally, the National Technical Council provides an assessment to the authority which authorizes or denies the request by means of a Motivated Resolution. GMO cultivation is usually monitored according to NTC recommendations (Colombia, 2005). 

Since 2004, Paraguay has grown GM crops and the area is currently dominated by soybeans at 83.1% of the country’s total GM area, followed by maize and cotton. In 2023, 4.3 Mhas of GM crops were grown (AgbioInvestor, 2024). The National Agricultural and Forestry Biosafety Commission (CONBIO) under the Ministry of Agriculture and Livestock oversees GMO risk assessment. The Commission is composed of representatives from the Ministry of Agriculture and Livestock (MAG - Ministerio de Agricultura y Ganadría), Ministry of Public Health and Social Welfare (MSPyBS - Ministerio de Salud Pública y Bienestar Social), Ministry of Industry and Commerce (MIC - Ministerio de Industria y Comercio), National Service for Plant and Seed Health and Quality (SENAVE - Servicio Nacional de Sanidad y Calidad Vegetal y de Semillas), National Service for Animal Health and Quality (SENACSA - Servicio Nacional de Sanidad y Calidad Animal), National Forestry Institute (INFONA - Instituto Forestal Nacional), Paraguayan Institute of Agricultural Technology (IPTA - Instituto Paraguayo de Tecnología Agropecuaria), Ministry of Environment and Sustainable Development (MADES - Ministerio de Ambiente y Desarrollo Sostenible) and National University of Asunción (UNA - Universidad Nacional de Asunción). The market approval is granted by the Ministry of Agriculture and Livestock, based on the recommendations issued by CONBIO, which is also in charge of granting permits for the performance of GMO-confined activities (CONBIO, 2024).

Uruguay has cultivated GM crops since 2000 and in 2023 the country grew 1.2 Mhas of GM maize and cotton (AgbioInvestor, 2024). The entities in charge of authorizing all GMO-related activities in Uruguay are gathered in the National Biosafety System (SNB - Sistema Nacional de Bioseguridad). Within the SNB, the Biosafety Cabinet (GNBio- Gabinete de Bioseguridad) is the authority that regulates access to technologies, issuing regulations and advising GNBio in matters related to GMO risk assessment and management. This cabinet is composed of representatives of each of the six ministries: Agriculture Livestock and Fisheries, Public Health, Environment, Industry, Economy, and Foreign Affairs.

The GMO technical and scientific assessments are performed by the Biosafety Risk Assessment (ERB - Evaluacíon de Riesgos de Bioseguridad) entity, which coordinates a multidisciplinary team of experts and produces reports and technical recommendations for the CGR. The risk assessment comprises GMO’s risks for the environment, biological diversity, human, animal and plant health, as well as the social and economic impacts of the GMO. To coordinate all these tasks, ERB is supported by the Committee for Institutional Coordination (CAI - Comité de Coordinacíon Institucional) (Uruguay, 2024).

## Biosafety of GMOs in Brazil 

Over the last two decades, the total area cultivated with GM crops has greatly increased. From the initial planting of 1,7 Mhas in 1996 when the first biotech crop was commercialized, the 2019 planting indicates a ~112-fold increase. Among the countries producing GM crops, in the 2018/2019 crop season, Brazil had the second largest crop area, with more than 53 Mhas surpassed only by the USA with 71,5 Mhas (ISAAA, 2021). Soybean was and still is the main seeded GM crop in the country, with engineered cultivars corresponding to 97 and 98% of total production in crop seasons 2019/2020 and 2020/2021, respectively (Proterra Foundation, 2021). In 2023, Brazil cultivated 66.9 million hectares of GM cotton, maize, soybean and sugarcane (AgbioInvestor, 2024). 

In this context, and considering the continuous growth in GM fields, a clear and well-established regulatory framework is required to ensure human and animal health and environmental protection from the potential adverse effects of GMOs. Thus, Biosafety Law No. 11,105 was created on 24^th^ March 2005 and was important in establishing the guidelines for the safe use of genetic engineering technologies in research and development, keeping Brazil as an important contributor in the areas of agriculture, industry, and human/animal welfare (Details can be found at: http://ctnbio.mctic.gov.br/en/leis/-/asset_publisher/NT53w3Yb7zpx/content/lei-n-11-105-de-24-03-2005) (Nepomuceno et al., 2020). This law also created safety norms and inspection mechanisms for activities that involve genetically modified organisms (GMOs) and their by-products, as well as for construction, cultivation, production, handling, transportation, transfer, import, export, storage, research, environmental release, unloading, and commercialization (Nepomuceno *et al.*, 2020; Vieira et al., 2021). In addition, it implemented the National Biosafety Council (CNBS - in Portuguese Conselho Nacional de Biossegurança), restructured the National Biosafety Technical Commission (CTNBio - in Portuguese - Comissão Técnica Nacional de Biossegurança), and provided for the National Biosafety Policy (PNB - in Portuguese Política Nacional de Biossegurança) (CTNBio, 2022). A brief description of these committees and their role and importance in the biosafety of GMOs will be given below.

The CNBS is a collegiate body composed of eleven high representatives of the State, including the Chief of Staff of the Presidency, who presides over it. The Minister of Justice; Minister of Science, Technology and Innovations; Minister of Agricultural Development; Minister of Agriculture, Livestock and Supply; Minister of Health; Minister of the Environment; Minister of Development, Industry and Foreign Trade; Minister of Foreign Affairs; Minister of Defense, and the Secretary of Aquaculture and Fisheries complete the council. This council provides advisory assistance to the President of the Republic in the formulation and implementation of the National Biosafety Policy, establishing principles and guidelines that consider socio-economic and political conveniences, and opportunities of national interest involved in the commercial use of GMOs and related products. The technical opinion from CNBS members on a final decision to release a GMO for commercial use will only be requested if socio-economic and/or strategic policy decisions are required. It is CTNBio’s responsibility the technical judgment on the biosafety of a commercially used GMO. Nevertheless, CNBS has 30 days to refute the commercial approval of a GMO after CTNBio has released its official position. If the refutation does not occur within 30 days, the product is automatically authorized for sale (Nepomuceno et al., 2020; Vieira et al., 2021; CTNBio, 2022). 

The CTNBio is a multidisciplinary advisory and deliberative collegiate that provides assistance and technical support to the Federal Government to formulate, update, and implement the National Biosafety Policy for the development of GMO products or biotechnology products that at some stage could generate a GMO. This commission establishes technical safety standards, through Normative Resolutions, regarding the authorization of activities related to research, and the commercial release of GMOs and it is also responsible for zoo-sanitary, phytosanitary, human health, and environmental risks assessment establishing risk management measures as well. Other competencies of CTNBio include authorizing the importation of GMOs for research, providing technical assistance to registration and inspection organizations, and monitoring the development and technical-scientific progress achieved in biosafety, biotechnology, bioethics, and related areas, with the aim to increase the capacity of protecting human, animal and plant health, and the environment. 

The CTNBio’s president and its 27 full members and their substitutes are appointed by the Minister of Science, Technology and Innovations for a 2-year term, extendable for the same period. They must be Brazilian citizens with recognized technical competence and outstanding participation in the scientific community. All members must have a doctorate, and be professionally active in the areas of biosafety, biotechnology, biology, microbiology, health and environment, human/animal health, or related areas. Twelve members of the scientific community are directly appointed by the Minister, while the others are appointed by one of the bodies of the CNBS: Ministry of Agriculture, Livestock, and Supply; Ministry of Health; Ministry of the Environment; Ministry of Agricultural Development; Ministry of Development, Industry and Foreign Trade; Defense Ministry; Secretariat of Aquaculture and Fisheries; Ministry of Foreign Affairs, and Ministry of Justice. The complete list of CTNBio’s members can be found on its website. 

The commission is organized into permanent sectoral sub-commissions in the areas of plant and environment, human, and animal health and it has a permanent executive secretariat that provides technical and administrative assistance and organizes monthly meetings (except in January and July). The meetings can be held with the 14-member quorum (half plus one), including at least one representative from each of the four subcommittees. If necessary, representatives of the scientific community, the public sector, and civil society entities with experience in a specific field may be invited to attend meetings, but they are not entitled to vote. Any decision taken by CTNBio must be approved by a majority vote. To provide greater transparency to the process, all decisions are published in the official journal and open for public comment within 30 days. In the same way, all meetings are open to citizens, who can consult the agendas, as well as all documents produced by the commission, which are available on the CTNBio’s website (http://ctnbio.mctic.gov.br/en/a-ctnbio) (Nepomuceno et al., 2020; Vieira et al., 2021; CTNBio, 2022). 

Besides ensuring biosafety, CTNBio is also the commission issuing permission to anyone interested in carrying out activities with GMOs. Among its prerogatives, the law delegates to CTNBio the assessment of new technologies and their possible impacts on the environment, and human and animal health in the country. It is also CTNBio that oversees the regulatory framework of the collective known as New Breeding Technologies (NTBs). All guidelines and technical requirements for the presentation consultation to CTNBio on products derived from these techniques can be found in Normative Resolution N. 16 (RN16), published on 15^th^ January 2018. Briefly, this document determines, through a case-by-case consultation system, whether a product generated by NBTs should or not be classified as GMO. For this consultation, the developer institution must provide information about the original organism and the product, including the methods used to generate it, and its molecular analysis. In practical terms, products obtained by random mutation directed to the site that involves the non-homologous end joining (NHEJ) (considered SDN1- site direct nuclease- mutation) or homologous repair directed (HRD) to the site that involves few nucleotides (SDN2 mutation) and that meet the conditions established in RN16, could be designated as non-GMO. In contrast, transgene inserts targeted to the site (SDN3 mutation) will probably be classified as GMO, according to the RN16. If the product is designated as GMO, the developer must comply with all biosafety requirements and will be approved only after CTNBio’s risk assessment. The product can be registered using existing procedures if it is not classified as a GMO. RN16 applies to all types of organisms, including plants, animals, and microorganisms, in the research and/or commercial release phase (Vieira et al., 2021, CTNBio, 2022). 

Finally, besides all the above-cited committees, every University, Research Institute, and company, either public or private that is developing research with GMOs, must have an Internal Biosafety Commission (CIBio -in Portuguese Comissão Interna de Biossegurança) which is a local biosafety committee, usually composed of individuals with adequate training and education in the areas of biotechnology, genetic engineering, biosafety, or other related fields. In addition, all public and private organizations, national or foreign, that want to carry out research activities or projects in Brazil, must request the Certificate of Quality in Biosafety (CQB - in Portuguese Certificado de Qualidade em Biossegurança) issued by CTNBio before starting any activity. As a mandatory procedure, for every research/project using such technologies at the Institution/University/companies, a principal investigator must be indicated as responsible. Furthermore, each CIBio is legally responsible for ensuring the biosafety conditions of the entity facilities, performing regular audits, and sending an annual report of its activities and projects to the CTNBio.

Another important component of the Brazilian Regulatory Framework is the Organizations and Entities of Registration and Inspection (OERF) which includes the Ministry of Agriculture, Livestock and Food Production, the Ministry of Health, the Ministry of Environment, and the Ministry of Aquaculture and Fisheries. Under Law No. 11.105 and within their field of competence, in compliance with CTNBio technical resolutions and technical opinions, the OERF are responsible for monitoring GMOs and their by-products. The responsibilities of OERF include inspecting research activities, registering and inspecting the commercial use of GMOs, granting authorization for importing products for research and commercial use, and keeping updated information regarding institutions and principal investigators that carry out activities and projects. All OERF also assist CTNBio in defining biosafety assessment parameters, disclosing to the public, grant registrations and authorizations for the commercial use of GMOs, enforcing the law and applying the established penalties when noncompliance is identified (Nepomuceno et al., 2020; Vieira et al., 2021; CTNBio, 2022).

## Genetic engineering for tolerance to biotic conditions - the research phase

Considering the importance of biotic challenges in the field, some Brazilian researchers have focused on developing soybean lines showing tolerance to these conditions, from fungi to caterpillars and nematodes. Although most of these studies are in progress, a brief description of these works will be presented following. 

Homrich et al. (2008 a ) developed genetically modified (GM) soybean lines using particle bombardment, which contained a truncated synthetic *cry1Ac* gene from *B. thuringiensis*. *In vitro* insect feeding assays showed that these transgenic plants were highly toxic to the velvetbean caterpillar (*Anticarsia gemmatalis*), a major soybean pest. In a separate study, eleven homozygous progenies expressing the *cry1Ac* gene were evaluated for agronomic characteristics compared to the non-transformed parent ‘IAS 5’ cultivar. The presence of the *cry1Ac* transgene did not have any negative effects on the yield and agronomic performance of the transgenic plants, considering the majority of analyzed traits. The transgenic plants exhibited a normal karyotype (2n = 40) with no apparent chromosomal abnormalities. Additionally, two out of the 11 transgenic progenies were evaluated *in vitro* and *in vivo* for resistance against *A. gemmatalis*. The bioassays included two negative controls: a transgenic homozygous line containing only the *gusA* reporter gene, and non-transgenic ‘IAS 5’ plants. In the detached-leaf bioassay, larvae reared on *cry1Ac* leaves showed 100% mortality after 72 hours, while only 1.65% of the larvae fed on non-transgenic leaves died. The whole-plant feeding assay confirmed the high protection provided by *cry1Ac* against velvetbean caterpillars. The percent defoliation of the homozygous *gusA* line and non-transgenic ‘IAS 5’ plants, fourteen days after the infestation, was, on average, 56.5 and 71.5%, respectively, while almost no damage was observed on *cry1Ac* transgenic progenies (Homrich *et al.*, 2008b).

Plant ureases are multifunctional enzymes that catalyze the enzymatic hydrolysis of urea into ammonia and carbon dioxide, thereby participating in nitrogen metabolism. In addition to their well-established role in nitrogen metabolism, several studies have demonstrated the toxic effects of ureases on fungi and insects. Among the various plant species, soybean possesses three distinct isoforms of urease: the ubiquitous urease encoded by the *Eu4* gene, the embryo-specific urease encoded by the *Eu1* gene, and the SBU-III urease encoded by the *Eu5* gene (Wiebke-Strohm et al., 2016). To investigate the involvement of the ubiquitous urease in soybean’s response to fungal infections, an initial approach involved transcript analysis and manipulation of *GmEu4* gene expression in transgenic soybean plants. Differential expression patterns of *GmEu4* were observed in susceptible and resistant soybean genotypes throughout the infection caused by *Phakopsora pachyrhizi*. 

Although the original intention was to overexpress *GmEu4*, the transgenic plants unexpectedly exhibited GmEu4 co-suppression and reduced ureolytic activity. However, these co-suppressed plants proved to be valuable tools for studying the function of *GmEu4*. Protein extracts derived from transgenic co-suppressed plants showed a significantly higher promotion of growth for *Rhizoctonia solani*, *Phomopsis* sp., and *Penicillium herguei*, compared to extracts from non-transgenic plants. Moreover, when detached leaves of transgenic plants were infected with *Phakopsora pachyrhizi* uredospores, they exhibited a significantly greater number of lesions, pustules, and erupted pustules compared to leaves from non-transgenic plants. These results suggest that soybean plants lacking urease are more susceptible to fungal infections. Considering the challenges associated with urease overexpression in its intact form, the authors proposed an alternative approach involving the identification and overexpression of the urease fungitoxic peptide (Homrich et al., 2012).

Research has also been conducted on nematode resistance. Soybean ‘composite’ plants (i.e. plants consisting of wild-type shoots and transgenic hairy roots) and whole-transgenic plants overexpressing a peptide named Soyuretox, derived from the soybean ubiquitous urease, were produced by *Agrobacterium rhizogenes* and bombardment transformation, respectively. Two bioassays were conducted to evaluate the potential of Soyuretox in conferring resistance against nematodes. In the first bioassay, ‘composite’ plants were inoculated with *Meloidogyne javanica* J2 larvae. Hairy roots overexpressing Soyuretox exhibited a significant reduction (48%; p < 0.05) in the nematode reproductive factor compared to roots transformed with an empty vector. In the second bioassay, whole transgenic plants overexpressing Soyuretox and challenged with *M. javanica* also showed a significant reduction (37.5%; p < 0.05) in the nematode’s reproductive factor compared to non-transformed plants. These results suggest that Soyuretox can confer resistance to root-knot nematodes in soybeans, making it a potential candidate for integrated pest management systems against this important parasite and possibly other plant parasitic nematodes (Fraioli et al., 2019).

As for fungal diseases in soybean, Sclerotinia stem rot (SSR) and Asian soybean rust (ASR) are among the most important in the Brazilian scenario. Due to that, these diseases are the focus of research aiming to develop lines resistant lines, to minimize losses in production, as the presence of these fungi increases the costs for crop maintenance and disease control. In this context, aiming to develop soybean lines resistant to *S. sclerotiorum,* Cunha et al. (2010) introduced the oxalate decarboxylase (*oxdc*) gene from a *Flammulina* sp. in a commercial soybean variety using biolistic. Previous studies had demonstrated that oxalic acid (OA) was involved in *S. sclerotiorum* pathogenesis and that plants more tolerant to OA were also more tolerant to the fungus (Guimarães and Stotz, 2004; Walz et al., 2008). Results from the disease progress curve showed a significant delay in the symptom development in all *oxdc* events when compared to the non-transgenic background. Some plants did not present any symptoms after 92 h of infection, suggesting immunity, and demonstrating that the *oxdc* gene may play an important role in the resistance to *S. sclerotiorum* in soybean (Cunha *et al.*, 2010). 

Seeking the development of soybean more tolerant to *P*. *pachyrhizi*, resistance gene *CcRpp1* (*Cajanus cajan* Resistance against *Phakopsora pachyrhizi* 1) from pigeon pea (*Cajanus cajan*) was introduced in soybean. Results from inoculation assays demonstrated that *CcRpp1* homozygous soybean lines displayed high levels of resistance, with no visible lesions; hemizygous plants showed RB type resistance, 60-71% reduction in lesion count per cm^2^ and null plants presented tan-colored lesions and high sporulation (like susceptible phenotype). This study demonstrated that it is possible to effectively transfer a dominant resistance gene from a related legume into soybean plants and obtain a high level of resistance to ASR (Kawashima et al., 2016).

## Genetic engineering for tolerance to abiotic conditions 

Among all the abiotic conditions that soybean crop is exposed to, drought, heat and excess rainfall are the ones that most impact grain growth, development, and final yield in the world as well as in Brazil. Especially water deficit is the factor that most causes production losses. During the period between the 2003/2004 and 2014/2015 crop seasons, losses due to this environmental adverse condition were estimated to be in the US$46,6 billion range (Fuganti-Pagliarini et al., 2017). Across a longer term (five decades), losses due to drought events are estimated at 11.65% of national soybean production, totaling ~280 million tonnes or US$152 billion (2022 base price). For instance, in the 2021/22 crop season, losses were ~23.8% (~30 million tonnes, ~US$16.3 billion); and even in the record 2022/23 season, yield losses reached ~6.3%, mostly in Rio Grande do Sul (Ferreira et al., 2024).

Extreme events such as drought and heat waves continue to cause substantial losses to Brazilian agribusiness. For instance, the severe drought in the 2021/22 season led to losses of about R$ 45.3 billion, while subsequent events associated with El Niño in 2023/24 also impacted soybean production, particularly in southern states (Ferreira et al., 2024). Due to this hostile environmental condition, the productivity estimate for soybean crop was revised from 56,38 bags per hectare to 53,69, a retraction of 14,56% in relation to the forecasted harvest, which is equivalent to 1.04 million tons. Accompanying this scenario of yield losses, financial ones are also expected. Pursuing alternatives to minimize damages from abiotic stresses scientists are developing plants more tolerant to either one or more than one condition, by introducing genes that can confer tolerance. Among them, responsive transcription factors (TFs) are a choice and are being introduced into soybean cultivars to obtain more adapted and tolerant lines. Among these TFs, genes from ABA-independent and dependent drought response pathways such as DREB (dehydration responsive element binding) (Polizel et al., 2011; Engels et al., 2013; De Paiva Rolla et al., 2014; Marinho et al., 2021) and AREB (ABA-responsive element-binding) (Barbosa et al., 2013; Marinho *et al.*, 2016), respectively, are shown to be an interesting option (Fuganti-Pagliarini, et al., 2017; 2020).

Many studies have shown the potential of the DREB family in improving drought tolerance in soybean. Under greenhouse (GH) conditions, events overexpressing AtDREB1A exhibited higher stomatal conductance, photosynthetic and transpiration rates, as well as increased chlorophyll content (Polizel et al., 2011). Data also suggested that the higher survival rates of DREB plants were related to reduced water use due to lower transpiration under well-watered conditions. When tested under field conditions in the 2011/2012 crop season, across four water regimes (irrigated, natural drought, and rain-out shelters at vegetative or reproductive stages), transgenic lines showed higher yield components particularly when drought occurred during the vegetative stage (De Paiva Rolla et al., 2014).

In subsequent crop seasons (2013/2014 and 2014/2015), DREB1 lines were again evaluated under the same conditions. Results indicated similar performance between GM and wild-type plants regarding chlorophyll content, plant height, and internode length under both irrigated and non-irrigated treatments. Oil and protein contents were not significantly affected in transgenic lines; however, in 2014/2015 protein levels were slightly higher and oil levels lower compared with the 2013/2014 season.

Molecularly, TF DREB1A induced the expression of many genes by binding to an essential cis-element with the core sequence A/GCCGAC named the dehydration responsive element (DRE) (Maruyama et al., 2004), present in the promoter region of drought-responsive genes. Thus, soybean endogenous drought-responsive genes such as *GmPI-PLC* (phospholipase C), *GmSTP* (sorbitol transporter protein), *GmGRP* (glycine-rich RNA-binding protein), and *GmLEA14* (late embryogenesis abundant), were highly expressed in GM plants submitted to severe water deficit stress (2.5% GH for approximately 30 days) in greenhouse experiments (Polizel et al., 2011). In field conditions, similar results were obtained. GM lines showed high *AtDREB1A* gene expression with a value reaching 4.806x. Compared to the WT line, DREB1A plants showed higher expression levels for putative soybean aquaporin pip1/UDP galactose transporter (Glyma12g066800) and alanine aminotransferase *GmAlaAT* (Glyma01g026700) genes. Yet, galactinol synthase/*Gols* (Glyma10G145300), involved in osmotic adjustment (OA) presented higher expression in the *AtDREB1A* GM line suggesting that this line might be targeting the OA mechanism to cope with water deficit (Fuganti-Pagliarini et al., 2017, 2020). 

Besides DREB1A TF, DREB2A is another gene being applied to develop soybean more drought tolerant. The overexpression of constitutively active (CA) DREB2A resulted in significant tolerance to drought and heat stress in transgenic *Arabidopsis* plants (Sakuma et al., 2006a, b), as well as other plant species (Dubouzet et al., 2003; Qin et al., 2007; Liu et al., 2008; Almoguera et al., 2009;). In soybean, *GmDREB2A;2* (Mizoi et al., 2013) was inserted into a commercial variety using biolistic. Expression assay results from leaves and roots submitted to various dehydration treatments under a hydroponics system, showed a high expression of the transgene, with the roots showing the highest expression levels during water deficit. As for physiological parameters, photosynthesis rate, stomatal conductance, transpiration, and leaf-air temperature difference in *AtDREB2A* CA GM plants contrasted from those of the non-GM control cultivar for each treatment, confirming the induction of the water deficit as well as indicating that plants overexpressing *AtDREB2A* and *DREB2A*-like proteins could increase tolerance to abiotic stress, such as drought and heat, which often occur together under field condition (Engels et al., 2013). In field experiments, the *AtDREB2A* line showed similar behaviour to WT plants in relation to chlorophyll content as well as regarding plant height and the mean length of internode in irrigated and non-irrigated treatments (Fuganti-Pagliarini et al., 2017, 2020). Like *DREB1A* lines, in *DREB2A* plants oil and protein contents were not affected, and values for protein content were higher and for oil content lower in crop season 2014/2015 when compared to crop season 2013/2014 (Fuganti-Pagliarini *et al.*, 2017, 2020). 

Yet, in field conditions, transgene expression in *AtDREB2CA* lines was 2.91x. As for the expression of drought-induced genes, the relative expression level of phosphatase *GmPP2C* (Glyma14g195200), chlorophyll a/b binding protein - *Cab21* (Glyma16g165800), alanine aminotransferase *GmAlaAT* (Glyma01g026700), putative soybean aquaporin pip1/UDP galactose transporter (Glyma12g066800), and putative soybean aquaporin pip2/aquaporin transporter/glycerol uptake facilitator (Glyma12g172500) genes was higher in *DREB2A* lines than in WT non-GM background (Fuganti-Pagliarini et al., 2017, 2020).The involvement of the TF DREB2A in water deficit responses at different developmental stages was also evaluated in soybean. Under osmotic stress during germination, seedlings from the GmDREB2A event showed less growth reduction compared to WT plants. When water deficit was applied during the vegetative (VEG) stage, GmDREB2A plants performed better in growth and physiological parameters, also maintaining higher green foliar area values after 19 days of withheld irrigation followed by eight days of rehydration. In greenhouse conditions, during the reproductive stage, GM lines tended to show higher yield components compared to other genotypes and treatments, suggesting superior performance associated with higher expression levels of the cisgene and drought-induced genes.

At the molecular level, under water deficit, GM plants exhibited higher expression of LEA proteins (LEA2 - Glyma.09G185500 and LEA6 - Glyma.17G16420) and the heat shock protein HSP70 (Glyma.17G072400), confirming the induction of drought-responsive pathways. Overall, these findings suggest that the enhanced performance of GmDREB2A lines during germination, emergence, and the vegetative stage may lead to better plant establishment and consequently improved yield stability, or reduced losses, in soybean production regions of Brazil (Marinho et al., 2021).

As mentioned above, ABA-dependent TFs were also used to develop drought-tolerant soybean (Barbosa et al., 2013; Leite et al., 2014; Marinho et al., 2016; Fuganti-Pagliarini et al., 2017, 2020; Molinari et al., 2023). Soybean GM lines overexpressing the AREB gene from A. thaliana (AtAREB1) were first generated by biolistics, resulting in low transgene copy numbers and higher expression levels. These lines were considered drought tolerant under greenhouse conditions, as they were able to grow and survive a five-day water deficit (WD) period, showing no leaf damage after rewatering (Barbosa *et al.*, 2013). Later, AtAREB1 lines obtained via Agrobacterium tumefaciens transformation also displayed superior performance. In greenhouse assays, differences were observed in root dry matter (RDM), leaf blade dry matter (LBDM), and total leaf area (LA) when comparing well-watered and WD treatments. Improved performance in transgenic lines relative to WT was likely associated with mechanisms of drought prevention, such as reduced stomatal conductance and transpiration under control conditions.

At the molecular level, under WD conditions, besides the expression of the transgene AtAREB1, GM lines showed ~20-fold higher expression of GmRAB18, a drought-responsive gene encoding a glycine-rich hydrophilic protein (Marinho et al., 2016). Another construct, an activated form of AtAREB1 (AREB1ΔQT), was also overexpressed in soybean, resulting in greater water use efficiency and more leaves compared to non-GM isolines. These plants showed smaller leaf area and higher stomatal conductance under well-watered conditions, likely reducing transpiration and delaying stress onset. Differences in wilting degree and survival rate between 35S-AREB1ΔQT and WT plants were also associated with regulation of dehydration-protective genes, including PP2C (Glyma14g37480), GmSRK2 kinase (Glyma02g15330), and GmRAB18 (Glyma09g31740) (Marinho *et al.*, 2016).

Soybean AREB lines were also assayed in real field conditions. Results showed that in crop season 2013/2014, *AtAREB1A* line presented higher intrinsic water use, leaf area index and plant height when compared to other genotypes and its background. As for yield, the *AtAREB1* line presented a higher number of nodes, a higher number of pods per plant, and the highest yield value, reaching 2.153 kg ha^-1^. Molecularly, when compared to other genotypes and WT plants, the *AtAREB1A* line presented higher expression levels of genes such as phosphatase *GmPP2C* (Glyma14G195200), alanine aminotransferase *GmAlaAT* (Glyma01G026700, and Glyma07G045900), Δ- 1-pyrroline-5-carboxylate synthetase - *P5CS* (Glyma18G034300), dehydrins (Glyma09G185500), heat shock proteins (Glyma17G072400), putative soybean aquaporin pip1/UDP galactose transporter (Glyma12G066800) and chlorophyll a/b binding protein - Cab21 (Glyma16G165800); all related to drought responses. In addition, as for protein and oil contents, in both crop seasons assayed in the field (2013/2014 and 2014/2015), line *AtAREB1A* showed the highest protein content and the lowest oil content, both in IRR and NIRR conditions, relative to the other plant materials, reaching values for protein content of around 41-42% and between 15 and 17% for oil content (Fuganti-Pagliarini et al., 2017). 

All data presented here for soybean GM lines containing TFs confirm that by expressing these genes and endogenous drought-responsive genes. Moreover, these plants can modulate their metabolism in response to water deficit conditions by targeting different mechanisms, aiming to survive and retain productivity as well as by keeping protein and oil contents market standards, to ensuring possible commercial cultivars derived from these lines competitiveness in the livestock market (Fuganti-Pagliarini et al., 2017). 

Still, in the category of TF, *NAC* (an acronym for NAM- no apical meristem, ATAF- *Arabidopsi*s transcription activation factor, and CUC- cup-shaped cotyledon) gene was overexpressed in soybean aiming for drought tolerance. Results showed that the photosynthetic activity and apparatus were more affected by drought in the transgenic lines. Consistent with hypersensitivity to drought, chlorophyll loss, and lipid peroxidation were higher in the *GmNAC81*-overexpressing lines than in the non-GM background under dehydration. These authors suggested that in addition to promoting stress-induced cell death, *GmNAC81* gene directly regulates drought-responsive genes and increases drought sensitivity, negatively controlling water deficit tolerance not only via vacuolar processing enzyme (VPE) activation, the executioner of the cell death program, but also via suppression of ABA signaling (Ferreira et al., 2020).

Despite TFs, other water deficit-responsive genes such as Gols (Honna et al., 2016) and NCED (Molinari et al., 2020) have also been introduced into soybean with the aim of improving drought tolerance. The enzyme galactinol synthase (GolS, EC 2.4.1.123) catalyzes the first step in raffinose family oligosaccharides (RFOs) biosynthesis, compounds that accumulate in plant tissues during stress and help stabilize membranes and maintain vital functions. Soybean events overexpressing Gols showed increased galactinol transcripts, acting as osmoprotectants and resulting in greater drought tolerance and higher yield in field trials (Honna *et al.*, 2016). These findings support the use of Gols as an additional tool in soybean breeding for drought tolerance.

The *NCED* gene encodes 9-cis-epoxycarotenoid dioxygenase (EC 1.13.11.51), a key enzyme in abscisic acid (ABA) biosynthesis. ABA plays a central role in drought responses by triggering physiological adjustments and activating defense-related genes. GM soybean lines overexpressing *NCED* showed elevated expression of drought-responsive genes such as *GmAREB1*, *GmPP2C*, *GmSnRK2*, and *GmAAO3* under stress, along with improved physiological performance and higher yield (Molinari et al., 2020). These results demonstrate that NCED is a promising candidate for incorporation into breeding programs targeting cultivars better adapted to dry environments. Another gene option that can be applied in GM strategy development is the chaperones family, molecules involved in facilitating the folding and assembly of proteins, protein transport across membranes, inhibition of misfolding and prevention and/or reversion of proteins aggregation. Using an endoplasmic reticulum (ER)-resident molecular chaperone BiP (binding protein), Valente et al. (2009) developed transgenic lines overexpressing the *soyBiPD* gene. Results showed that under drought, leaves in the transgenic lines did not wilt and exhibited a smaller decrease in water potential. Rates of photosynthesis and transpiration were less inhibited in GM than in WT plants. In addition, several typical drought-induced genes had their mRNA levels increased, and leaf senescence was delayed in GM lines, showing that *BiP* overexpression confers tolerance to drought. 

Besides the involvement of *Bip* genes in water deficit tolerance, some authors described, based on their results, its participation in the acceleration of the hypersensitive response (HR), potentially being a candidate for metabolic engineering of plant resistance against pathogens. Data from Rodrigues et al. (2021) showed that transgenic plants presented a lower abundance of HR-responsive metabolites when compared to infected WT plants. In contrast, salicylic acid (SA) biosynthetic and signaling pathways were more expressed in transgenic plants, as well as pathogenesis-related genes (PRs) and transcriptional factors controlling the SA pathway. In addition, *GmNAC32* transcriptional factor was more induced in the transgenic plants, and it has also been reported to regulate flavonoid synthesis in response to SA. In fact, the *BiP*- overexpressing plants demonstrated an increase in flavonoids, mainly prenylated isoflavones, as precursors for phytoalexins. 

Another successful application of a non-transcription factor (non-TF) gene in developing soybeans tolerant to water deficit was reported by Weber et al. (2014). In this study, a gene encoding an osmotin-like protein (SnOLP) from Solanum nigrum var. americanum was introduced into the soybean genome under the *Arabidopsis thaliana* UBQ3 promoter using particle bombardment. Osmotin-like proteins (OLPs) are produced by plants in response to various abiotic and biotic stresses and are associated with antifungal activity and osmotic stress tolerance (Ullah *et al.*, 2018). Two independent transgenic soybean lines (B1 and B3) expressing SnOLP were obtained, showing no significant phenotypic alterations over seven generations.

Under greenhouse water deficit conditions, transgenic plants performed better than wild-type (WT) controls. Physiological analyses revealed that transgenic plants maintained higher predawn leaf water potential, CO₂ assimilation rate, stomatal conductance, and transpiration compared to WT. Despite these advantages, both GM and WT plants experienced reductions in grain yield and 100-grain weight when stress was applied at the beginning of pod formation (R3 stage), with losses mainly attributed to pod abortion. However, the decrease in grain production was more pronounced in WT plants (approximately 20%) compared to transgenic plants (4.5% and 5.3% for B1 and B3 lines, respectively). Under well-watered conditions, 100-grain weight was similar for transgenic and WT lines. However, under water stress, 100-grain weight decreased by approximately 12% in WT plants, whereas it decreased by approximately 1.1% (B1) and 3.7% (B3) in transgenic plants. Furthermore, B1 and B3 lines exposed to water shortage exhibited significantly higher 100-grain weights than WT plants (p < 0.05). Overall, these results demonstrate that the expression of the *SnOLP* gene in soybeans improved physiological responses and yield components under water deficit conditions, highlighting the potential of this gene for biotechnological applications (Weber et al., 2014).

Although most of the papers focus on developing soybean GM lines more tolerant to drought which is the most concerning abiotic condition in Brazil and worldwide, some research on waterflooding is undergoing. As known water stress ranks as one of the major limiting factors for species distribution and crop production worldwide. In both conditions either drought or waterlogging, a decrease in energy production hampers primary and secondary metabolisms, affecting plant growth, development, and yield (Koltun et al., 2022). Plant hemoglobins are proteins involved in reactive oxygen species (ROS) concentration modulation produced under stressful conditions such as water disruptions. The role of these proteins was assayed in soybeans. *GmGLB1-1* and *GmGLB1-2* genes were moderately and highly expressed respectively, under waterlogging. Plants with roots overexpressing *GmGLB1-*1 or *GmGLB1-2* (mostly) showed higher transcript abundance of stress-defensive genes involved in anaerobic, nitrogen, carbon, and antioxidant metabolism when submitted to waterlogging. In addition, *GmGLB1-2* soybean presented lower H_2_O_2_ root content, and reactive oxygen species, under water excess compared to the control conditions. These authors suggested based on the results obtained that the *GmGLB1-2* gene is a strong candidate for soybean genetic engineering to generate waterlogging-tolerant soybean cultivars (Koltun *et al.*, 2022). 

It’s important to highlight that all information presented here is in a research phase, and none of these events are approved for commercialization. 

## Genetic engineering of soybean as bio-factories 

To fulfill the increasing demand for new drugs in ever-bigger quantities, researchers have learned, by applying genetic engineering tools, to use plants as small drug “factories”, reducing production costs, and guaranteeing higher safety standards. This approach is called Plant Molecular Farming. Thus, transgenic plants expressing higher concentrations of the desired chemical, with no-negligible implications for the sustainability of the product overall, especially in economic and environmental terms were developed. In this context, Cunha et al. (2011a) generated genetically engineered soybean seeds expressing human growth hormone (hGH). By introducing the alpha prime (a0) subunit of b-conglycinin tissue-specific promoter from soybean and the a-Coixin signal peptide from *Coix lacryma-jobi*, into soybean, expression levels of bioactive hGH up to 2.9% of the total soluble seed protein content (corresponding to approximately 9 g kg^-1^) were measured in mature dry seeds. Results of ultrastructural immunocytochemistry assays indicated that the recombinant hGH was efficiently directed to bioassays showing that the hGH expressed was fully active. These data demonstrated that the use of tissue-specific regulatory sequences is an attractive and viable possibility for achieving high-yield production of recombinant proteins in stable transgenic soybean seeds.

In addition, the expression and stable accumulation of human coagulation factor IX (hFIX) in transgenic soybean seeds has also been reported. Using biolistic, researchers introduced in soybean embryonic axes, the hFIX gene under the transcriptional control of the a0 subunit of a b-conglycinin seed-specific promoter and an a-Coixin signal peptide. The hFIX protein was expressed at levels of up to 0.23% (0.8 g kg-1 seed) of the total soluble seed protein in the transgenic seeds. Protein extracts from transgenic seeds showed a blood-clotting activity of up to 1.4% of normal plasma. Researchers also reported that the correct processing and stable accumulation of functional hFIX in soybean seeds allowed stored for 6 years under room temperature conditions (Cunha et al., 2011b ).

Another case of soybean use as bio-factories is the production of biologically active recombinant cyanovirin-N (rCV-N), an anti-HIV protein in seeds. Results showed that pure, biologically active rCV-N was isolated with a yield of 350 µg/g of dry seed weight. The observed amino acid sequence of rCV-N matched the expected sequence of native CV-N and purified protein from soybean was active in anti-HIV assays. More interestingly, the standard industrial processing of oil extraction did not reduce the antiviral activity of recovered rCV-N, allowing the use of industrial soybean processing to generate both oil and a recombinant protein for anti-HIV microbicide development (O’Keefe et al., 2015).

These few examples above of soybean being used as a bio-factory in researchers being developed in Brazil show that the grain is a viable option to produce recombinant proteins due to its high protein content, known regulatory sequences, efficient gene transfer protocols, and a scalable production system under greenhouse conditions, in addition to seed-based production of recombinant proteins be an efficient strategy to achieve the accumulation, correct folding, and increased stability of these recombinant proteins (Cunha et al., 2011b).

## Genetic engineering of soybean oil composition for biodiesel production

With energy needs and environmental concerns driving worldwide interest in developing fuels based on renewable sources of non-fossil fuels, the use of soybean for biodiesel production has proved to be an interesting option. Most plant oils are largely composed of five fatty acids, palmitate (16:0), stearate (18:0), oleate (18:1), linoleate (18:2) and linolenate (18:3), presenting high levels of polyunsaturated fatty acids, which decreases oil stability, affects the cetane number and limits their use as biofuels. From a compositional standpoint, biodiesel with a high monounsaturated fatty acid (oleate) content is desired for fuel performance such as ignition quality, nitrogen oxide (NOx) emissions and oxidative stability. Therefore, plant oils with high levels of oleic acid and low levels of palmitic acid are good candidates for developing high-performance biodiesel. Thus, aiming at metabolic engineering soybean to achieve industry biodiesel requirements, the *FAD2-1* and *FatB* genes were placed under the control of the 35SCaMV constitutive promoter, introduced to soybean embryonic axes by particle bombardment and down-regulated using RNA interference methodology. Results showed that the metabolically engineered soy plants exhibited a significant increase in oleic acid (up to 94.58%) and a reduction in palmitic acid (to <3%) in the seed oil content. No structural differences were observed between the fatty acids of the transgenic and non-transgenic oil extracts (Machado et al., 2014; Murad *et al.*, 2014). This study illustrated the potential of metabolic engineering soybean to fulfill future demand for new renewable sources of energy. In addition to traits directly related to agronomic performance, other soybean events were developed with a focus on modified seed composition, such as Plenish™, which presents an altered fatty acid profile with increased oleic acid content, and Treus™, designed to improve starch quality. Although their adoption has been limited, these technologies represent alternative applications of genetic engineering in soybean, particularly in the context of industrial uses such as biodiesel production.

## Soybean genetically modified events approved for commercialization versus being grown in Brazil and South America

In Brazil, there are to date 22 soybean GM events approved for commercialization by CTNBio and most of these events show either insect or herbicide resistance only or both traits combined. Among the other characteristics inserted in these GM varieties, nematode resistance, drought tolerance, an increase in the oleic acid level and a decrease in linoleic acid levels are also included. As for the holding companies, these GM soybean events are registered by Monsanto, Bayer, Down AgroSciences LLC, DuPont’s (Pioneer Hi-Bred International Inc, BASF, INDEAR, Verdeca and events that were developed in partnership between different developers. Although all these 22 events are approved for commercialization, only 18 events are effectively being grown in Brazilian fields (Table 1). The other 4 events approved by CTNBio express either resistance to nematodes, herbicides, and insects, or stacked traits (CTNBio, 2024). 

Regarding all other South American countries, Argentina has, according to ISAAA, 23 events approved but only 21 are being grown. Colombia, Paraguay, and Uruguay have respectively, 29, 13 and 8 events approved, but only 26, 11 and 7 events are being grown in fields (Table 1). Table 1 presents a summary of all events approved by each country’s respective regulatory agency and effectively being grown. Name of the event, code, trade name, developer, genetically modified trait, method of trait introduction, if a single event or stacked, and approved use (Food-Fo, Feed-Fe, and Cultivation-C) are also listed.

**Table 1-:** List of soybean genetically modified events being grown/commercialized, name, event code, trade name, developer, genetically modified trait, method of trait introduction and countries where events are commercially approved for Food (Fo), Feed (Fe), and Cultivation (C). The date of approval for each use is also presented. Data obtained from ISAAA.

Event Name	Event Code	Trade Name	Developer	GM Commercial Trait	Method of Trait Introduction	Argentina	Brazil	Colombia	Paraguay	Uruguay
A2704-12	ACS-GMØØ5-3	Liberty Link^®^ soybean	BASF	(Singular) Glufosinate herbicide tolerance	Microparticle bombardment of plant cells or tissue	2011 (Fo, Fe, C)	2010 (Fo, Fe, C)	2012 (Fe)	-	2012 (C)
A5547-127	ACS-GMØØ6-4	Liberty Link^®^ soybean	Bayer CropScience	(Singular) Glufosinate herbicide tolerance	Microparticle bombardment of plant cells or tissue	2011 (Fo, Fe, C)	2010 (Fo, Fe, C)	2012 (Fe)	-	2012 (C)
CV127	BPS-CV127-9	Cultivance	BASF	(Singular) Sulfonylurea herbicide tolerance	Microparticle bombardment of plant cells or tissue	2013 (Fo, Fe, C)	2009 (Fo, Fe, C)	2012 (Fo), 2011 (Fe)	2012 (C)	2014 (C)
DAS44406-6	DAS-444Ø6-6	Not available	Dow AgroSciences LLC	(Stacked) Glufosinate herbicide tolerance, Glyphosate herbicide tolerance, 2,4-D herbicide tolerance	Agrobacterium tumefaciens-mediated plant transformation	201 (Fo, Fe, C)	2015 (Fo, Fe, C)	2016 (Fo)	-	2017 (Fo, Fe)
DAS68416-4	DAS-68416-4	Not available	Dow AgroSciences LLC	(Stacked) Glufosinate herbicide tolerance, 2,4-D herbicide tolerance	Agrobacterium tumefaciens-mediated plant transformation	2017 (Fo, Fe)	2015 (Fo, Fe, C)	2016 (Fo)	-	-
DAS81419	DAS-81419-2	Not available	Dow AgroSciences LLC	(Stacked) Glufosinate herbicide tolerance, Lepidopteran insect resistence	Agrobacterium tumefaciens-mediated plant transformation	2016 (Fo, Fe, C)	2016 (Fo, Fe, C)	-	2019 (Fo, Fe)	-
DAS81419 x DAS44406	DAS-81419-2 x DAS-444Ø6-6	Conkesta Enlist E3™ Soybean	Dow AgroSciences LLC	(Stacked) Glufosinate herbicide tolerance, Glyphosate herbicide tolerance, Lepidopteran insect resistance, 2,4-D herbicide tolerance	Conventional breeding - cross hybridization and selection involving transgenic donor(s)	2016 (Fo, Fe, C)	2017 (Fo, Fe, C)	2016 (Fo, Fe)	2019 (Fo, Fe)	-
DBN9004	DBN-Ø9ØØ4-6	Not available	INDEAR	(Stacked) Glufosinate herbicide tolerance, Glyphosate herbicide tolerance	Agrobacterium tumefaciens-mediated plant transformation	2019 (C)	-	-	-	-
DBN9004 x DNB08002	DNB-Ø9ØØ4-3 x DNB-Ø8ØØ2-3	Not available	INDEAR	(Stacked) Glufosinate herbicide tolerance, Glyphosate herbicide tolerance, Lepidopteran insect resistance	Conventional breeding - cross hybridization and selection involving transgenic donor(s)	2023 (Fe, Fo)	-	-	-	-
DNB08002	DNB-Ø8ØØ2-3	Not available	INDEAR	(Stacked) Glufosinate herbicide tolerance, Lepidopteran insect resistance	Agrobacterium tumefaciens-mediated plant transformation	2022 (Fo, Fe, C)	-	-	-	-
DP305423	DP-3Ø5423-1	Treus™, Plenish™	DuPont (Pioneer Hi-Bred International Inc.)	(Singular) Sulfonylurea herbicide tolerance, Modified oil/fatty acid	Microparticle bombardment of plant cells or tissue	-	2018 (Fo, Fe)	2016 (Fo, Fe)	-	-
DP305423 x GTS 40-3-2	DP-3Ø5423-1 x MON-Ø4Ø32-6	Not available	DuPont (Pioneer Hi-Bred International Inc.)	(Stacked) Glyphosate herbicide tolerance, Sulfonylurea herbicide tolerance, Modified oil/fatty acid	Conventional breeding - cross hybridization and selection involving transgenic donor(s)	2015 (Fo, Fe, C)	2018 (Fo, Fe)	2016 (Fo, Fe)	-	-
DP356043	DP-356Ø43-5	Optimum GAT™	DuPont (Pioneer Hi-Bred International Inc.)	(Stacked) Glyphosate herbicide tolerance, Sulfonylurea herbicide tolerance	Microparticle bombardment of plant cells or tissue	-	-	2010 (Fo, Fe)	-	-
FG72 (FGØ72-2, FGØ72-3)	MST-FGØ72-2	Not available	BASF	(Stacked) Glyphosate herbicide tolerance, Isoxaflutole herbicide tolerance	Microparticle bombardment of plant cells or tissue	2018 (Fo, C)	2015 (Fo, Fe, C)	2016 (Fo)	-	-
FG72 x A5547-127	MST-FGØ72-2 x ACS-GMØØ6-4	Liberty Link^®^ GT27™	Bayer CropScience	(Stacked) Glufosinate herbicide tolerance, Glyphosate herbicide tolerance, Isoxaflutole herbicide tolerance	Conventional breeding - cross hybridization and selection involving transgenic donor(s)	2018 (Fo, Fe, C)	2015 (Fo, Fe, C)	2016 (Fo, Fe)	-	-
HB4	IND-ØØ41Ø-5	Verdeca HB4 Soybean	Verdeca	(Singular) Drought stress tolerance	Agrobacterium tumefaciens-mediated plant transformation	2015 (Fo, Fe, C)	2019 (Fo, Fe, C)	2022 (Fo)	2019 (Fo, Fe)	-
HB4 x GTS 40-3-2	IND-ØØ41Ø-5 x MON-Ø4Ø32-6	Not available	INDEAR	(Stacked) Glyphosate herbicide tolerance, Drought stress tolerance	Agrobacterium tumefaciens-mediated plant transformation	2018 (Fo, C)	2019 (Fo, Fe, C)	-	2019 (Fo, Fe)	-
MON87701	MON-877Ø1-2	Not available	Monsanto Company	(Singular) Lepidopteran insect resistance	Agrobacterium tumefaciens-mediated plant transformation	2016 (Fo, Fe, C)	-	2019 (Fo)	2018 (Fo, Fe)	-
MON87701 x MON89788	MON-877Ø1-2 x MON-89788-1	Intacta™ Roundup Ready™ 2 Pro	Monsanto Company	(Stacked) Glyphosate herbicide tolerance, Lepidopteran insect resistance	Conventional breeding - cross hybridization and selection involving transgenic donor(s)	2012 (Fo, Fe, C)	2010 (Fo, Fe, C)	2012 (Fo), 2011 (Fe)	2013 (Fo, C)	2012 (Fo, Fe, C)
MON87705	MON-877Ø5-6	Vistive Gold™	Monsanto Company	(Stacked) Glyphosate herbicide tolerance, Modified oil/fatty acid	Agrobacterium tumefaciens-mediated plant transformation	-	-	2014 (Fo), 2012 (Fe)	-	-
MON87751 x MON87701 x MON87708 x MON89788	MON-87751-7 x MON-877Ø1-2 x MON87708-9 x MON89788-1	Not available	Monsanto Company	(Stacked) Glyphosate herbicide tolerance, Modified oil/fatty acid, Dicamba herbicide tolerance	Conventional breeding - cross hybridization and selection involving transgenic donor(s)	-	2018 (Fo, Fe, C)	2018 (Fe)	2022 (Fo, Fe)	-
MON87705 x MON89788	MON-877Ø5-6 x MON-89788-1	Not available	Monsanto Company	(Stacked) Glyphosate herbicide tolerance, Modified oil/fatty acid	Conventional breeding - cross hybridization and selection involving transgenic donor(s)	-	-	2015 (Fo)	-	-
MON87708	MON-877Ø8-9	Genuity^®^ Roundup Ready™ 2 Xtend™	Monsanto Company	(Stacked) Glyphosate herbicide tolerance, Dicamba herbicide tolerance	Agrobacterium tumefaciens-mediated plant transformation	-	2016 (Fo, Fe, C)	2015 (Fo)	2019 (Fo, Fe)	-
MON87708 x MON89788	MON-877Ø8-9 x MON-89788-1	Not available	Monsanto Company	(Stacked) Glyphosate herbicide tolerance, Dicamba herbicide tolerance	Conventional breeding - cross hybridization and selection involving transgenic donor(s)	2018 (Fo, Fe)	eu tbm na	2012 (Fo, Fe, C)	-	2012 (C)
MON87708 x MON89788 x A5547-127	MON-877Ø8-9 x MON-89788-1 x ACS-GMØØ6-4	Not available	Monsanto Company	(Stacked) Glufosinate herbicide tolerance, Glyphosate herbicide tolerance, Dicamba herbicide tolerance	Conventional breeding - cross hybridization and selection involving transgenic donor(s)	-	-	2018 (Fe)	-	-
MON87712	MON-87712-4	Not available	Monsanto Company	(Singular) Glyphosate herbicide tolerance, Enhanced Photosynthesis/Yield	Agrobacterium tumefaciens-mediated plant transformation	-	-	2014 (Fo)	-	-
MON87751	MON-87751-7	Not available	Monsanto Company	(Singular) Lepidopteran insect resistance	Agrobacterium tumefaciens-mediated plant transformation	2018 (Fo, Fe), 2022 (C)	2017 (Fo, Fe, C)	2017 (Fo)	2019 (Fo, Fe)	-
MON87769	MON-87769-7	Not available	Monsanto Company	(Stacked) Glyphosate herbicide tolerance, Modified oil/fatty acid	Agrobacterium tumefaciens-mediated plant transformation	-	-	2014 (Fo), 2012 (Fe)	-	-
MON87769 x MON89788	MON-87769-7 x MON-89788-1	Not available	Monsanto Company	(Stacked) Glyphosate herbicide tolerance, Modified oil/fatty acid	Conventional breeding - cross hybridization and selection involving transgenic donor(s)	-	-	2015 (Fo)	-	-
MON89788	MON-89788-1	Genuity^®^ Roundup Ready 2 Yield™	Monsanto Company	(Singular) Glyphosate herbicide tolerance	Agrobacterium tumefaciens-mediated plant transformation	2016 (Fo, Fe)	-	2010 (Fo, Fe)	2018 (Fo, Fe)	2009 (C)
SYHT0H2	SYN-ØØØH2-5	Herbicide-tolerant Soybean line	BASF	(Stacked) Glufosinate herbicide tolerance, Mesotrione Herbicide Tolerance	Agrobacterium tumefaciens-mediated plant transformation	2017 (Fo, Fe, C)	-	2017 (Fo)	-	-

A brief description of all genes being used to develop these events, either approved in Brazil or South American countries, will be given in the following paragraphs. 

Under the category of herbicide resistance, the choices of molecules to develop soybean events from commercial use in Brazil were glufosinate (*pat* or *bar* gene), glyphosate (*2mepsps* and *cp4epsps* genes); sulfonylurea (*csr-1-2* and *gm-hra* genes), 2-4D (*aad-12* gene), and dicamba (*dmo* gene). 

The tolerance to glufosinate-based herbicides is conferred by the pat (also known as bar) gene, originally isolated from Streptomyces spp., which encodes the enzyme phosphinothricin acetyltransferase (PAT). This enzyme detoxifies the active L-isomer of phosphinothricin (*PPT*) by acetylation, producing N-acetyl-PPT, a non-toxic derivative that no longer inhibits glutamine synthetase (GS). Glufosinate ammonium itself is an equimolar, racemic mixture of the D- and L-isomers of PPT, but only the L-isomer is biologically active. As a glutamic acid analogue, L-PPT competes with the natural substrate of GS, an enzyme central to nitrogen assimilation. Its inhibition rapidly leads to ammonia accumulation in plant tissues, depletion of glutamine and other amino acid pools, and disruption of carbon metabolism. These effects indirectly impair photosynthesis and result in chlorosis, necrosis, and ultimately plant death within a few days (Schütte *et al.*, 2017).

The ammonium salt of L-PPT was first commercialized in the 1980s under the trade name Basta^®^ by Hoechst AG (later Aventis, now BASF). Since then, glufosinate has become one of the most widely used non-selective herbicides for crop and non-crop applications. Its activity is characterized by a broad spectrum of control over annual and perennial grasses and broadleaf weeds. Because glufosinate is primarily a contact herbicide with limited systemic translocation, effective field use requires good spray coverage of weed foliage. Although this limits its mobility compared to systemic herbicides, it provides a rapid necrotic response and can contribute to diversified weed management programs.

In soybean, glufosinate tolerance has been introduced both as a single trait and in stacked events. A well-known example is the LibertyLink^®^ technology, which relies on the bar/pat genes encoding phosphinothricin acetyltransferase, an enzyme that inactivates glufosinate, thereby conferring crop tolerance. LibertyLink^®^ soybeans were among the first commercial applications of this trait and are still present in several breeding programs, often as part of stacked events. A notable case is the Verdeca HB4^®^ soybean, which combines drought tolerance (through the *Hahb-4* gene from Helianthus annuus) with glufosinate resistance, approved in Brazil in 2019. Other commercial events combine pat/bar genes with insect resistance traits (Bt Cry proteins), providing simultaneous weed and pest control and expanding management flexibility for farmers. From an agronomic perspective, glufosinate-tolerant soybeans, including LibertyLink^®^ cultivars, represent an important tool for herbicide diversification and resistance management, ensuring broader sustainability of weed control practices. Although glufosinate is considered less persistent in the environment compared to several other herbicides and shows a favorable toxicological profile, continuous reliance on a single herbicide mode of action may still select for resistant biotypes. To ensure long-term effectiveness, best practices recommend integrating glufosinate into diversified weed control strategies, including herbicide mixtures, crop rotation, and cultural practices (Takano et al., 2020; Heap, 2025). Thus, glufosinate-tolerant soybean events not only broaden weed management options but also contribute to sustainable agricultural systems when combined with appropriate stewardship programs.

Glyphosate is the most widely used herbicide worldwide and has been central to modern agricultural systems since its introduction in the 1970s. Its broad-spectrum activity makes it lethal to a wide range of annual and perennial plant species, both grasses and broadleaves. The herbicide acts by inhibiting the enzyme 5-enolpyruvylshikimate-3-phosphate synthase (*EPSPS*), which catalyzes the conversion of 3-phosphoshikimate and phosphoenolpyruvate (*PEP*) into 5-enolpyruvylshikimate-3-phosphate, an essential step in the shikimate pathway. This pathway is responsible for the biosynthesis of the aromatic amino acids phenylalanine, tyrosine, and tryptophan, which are crucial for protein synthesis and also serve as precursors for numerous secondary metabolites. Inhibition of EPSPS by glyphosate leads to a rapid accumulation of shikimate intermediates, depletion of aromatic amino acids, metabolic disruption, and ultimately plant death (Patterson et al., 2018; Duke, 2018).

To generate glyphosate-tolerant crops, the most widely used strategy has been the insertion of the cp4-epsps gene and its variants, originally isolated from Agrobacterium sp. strain CP4. This bacterial *EPSPS* enzyme is naturally insensitive to glyphosate due to structural differences at the active site, allowing efficient catalysis even in the presence of high herbicide concentrations. When expressed in transgenic plants, CP4 *EPSPS* confers a high level of glyphosate tolerance, enabling herbicide application at rates sufficient to control weeds without damaging the crop (Padgette et al., 1995; Funke et al., 2006). Beyond CP4 *EPSPS*, additional biotechnological approaches have also been developed to enhance tolerance, such as the use of modified plant EPSPS enzymes with reduced glyphosate binding, and detoxification mechanisms involving glyphosate oxidoreductase (GOX) or glyphosate N-acetyltransferase (GAT).

In soybean, glyphosate-tolerant cultivars, often referred to as Roundup Ready^®^, were first commercialized in the mid-1990s and rapidly transformed weed management worldwide. The adoption of glyphosate-tolerant soybean simplified herbicide regimes facilitated no-till agriculture, and reduced the need for multiple herbicide applications. Stacked events have since been introduced, combining glyphosate tolerance with resistance to other herbicides such as dicamba or 2,4-D, as well as with insect resistance traits. These stacked traits increase flexibility in weed control programs and provide tools to manage resistant or hard-to-control species (Green, 2018).

Despite its success, the extensive reliance on glyphosate as a single mode of action has resulted in the evolution of glyphosate-resistant weeds, now documented in numerous species and across multiple continents (Heap, 2025). In response, integrated weed management strategies are strongly recommended, including herbicide rotation, mixtures with other modes of action, and non-chemical practices. Nevertheless, glyphosate-tolerant soybean remains a cornerstone of global agriculture due to its effectiveness, safety profile, and compatibility with conservation practices. It is important to highlight, however, that the evolution of tolerance to the widely used, nonselective herbicide glyphosate in weedy species endangers the continued success of GM crops glyphosate-resistant and the sustainability of glyphosate as the world’s most important herbicide. Indeed, past reliance on glyphosate and mistakes made in the stewardship of the glyphosate-resistant cropping system have directly led to the current weed resistance problems that now occur in many agronomic cropping systems (386 weed-resistance cases reported by 2025 - http://weedscience.org/summary/ResistByActive.aspx), showing the importance of well-stewarded the new technologies (Gage et al., 2019; Heap, 2025). 

In this context, another molecule used to control weed is the sulfonylurea (*csr-1-2* and *gm-hra* genes). The primary target of this herbicide group is the acetolactate synthase (ALS; EC 4.1.3.18) enzyme, which catalyzes the first common step in the biosynthesis of the branched-chain amino acids valine, leucine, and isoleucine (Lee et al., 1988). Because of its central role in amino acid metabolism, ALS is a highly effective herbicide target, and several chemical families act as potent ALS inhibitors. The most commercially relevant are the imidazolinones and sulfonylureas, followed by triazolopyrimidines, pyrimidinyl-thiobenzoates, and sulfonyl-aminocarbonyl-triazolinones (Mazur and Falco, 1989; Tranel and Wright, 2002). These herbicides disrupt protein biosynthesis, leading to growth arrest and eventual plant death, and they are valued in agriculture for their broad-spectrum weed control at low application rates. However, their intensive use has also resulted in the rapid evolution of resistant weed populations worldwide, making herbicide-tolerant crops a strategic tool to prolong their usefulness (Yu and Powles, 2014).

Amongst the ALS herbicides, Cultivance^®^ was a remarkable milestone for Brazilian soybean biotechnology. Resulting from a public-private partnership between Embrapa and BASF, this event was obtained by introducing the *csr1-2* gene from Arabidopsis thaliana, encoding a modified acetohydroxyacid synthase large subunit (*AtAHASL*) that confers tolerance to imidazolinone herbicides (ISAAA, 2021). Approved in 2009, Cultivance was the first genetically modified soybean developed in Brazil, representing an important achievement for national science and providing an alternative post-emergence weed control system. Nevertheless, despite its scientific relevance, Cultivance adoption remained limited due to multiple factors: the market dominance of glyphosate-tolerant soybeans, the carryover issues and pre-existing resistance associated with imidazolinone herbicides, and the restricted commercial diffusion of the event compared to competing technologies. As a result, the event was eventually discontinued from active promotion, highlighting how the success of GM traits depends not only on technical feasibility but also on economic competitiveness, agronomic stewardship, and farmer adoption dynamics.

When commercially released in 1946, 2,4-dichloro phenoxyacetic acid (2,4-D) was one of the first synthetic herbicides introduced into agriculture and it revolutionized weed science by providing an efficient, selective, and low-cost chemical control method for broadleaf weeds. As a synthetic auxin herbicide (SAH), 2,4-D mimics the naturally occurring plant hormone indole-3-acetic acid (IAA), disrupting auxin signaling pathways and leading to uncontrolled cell division, abnormal vascular differentiation, and ultimately plant death (Grossmann, 2010). Its efficacy, combined with broad-spectrum activity, made 2,4-D one of the most widely used herbicides in the world and a cornerstone in weed control programs. More recently, 2,4-D tolerance was incorporated into soybean through the Enlist^®^ technology, which stacks tolerance to 2,4-D with glyphosate and glufosinate (Enlist E3^®^), and, in advanced versions such as Conkesta Enlist E3^®^, also includes insect resistance, expanding weed management options for growers.

Dicamba (3,6-dichloro-2-methoxybenzoic acid), another important auxinic herbicide, shares a similar mode of action with 2,4-D but exhibits different physicochemical properties and weed control spectra. Dicamba induces unregulated gene expression associated with auxin responses, resulting in excessive ethylene and abscisic acid accumulation, epinasty, and inhibition of photosynthetic processes, which culminate in plant growth arrest and necrosis (Behrens et al., 2007). Because of its relatively low cost and strong effectiveness against a wide range of broadleaf weeds, dicamba has become a critical tool in modern weed management.

The incorporation of auxinic herbicide tolerance into soybean has been achieved by the expression of detoxification enzymes that inactivate or metabolize these herbicides. For instance, the aad-12 gene from *Delftia acidovorans* encodes an aryloxyalkanoate dioxygenase that degrades 2,4-D, conferring crop tolerance (Wright et al., 2010). Similarly, tolerance to dicamba was developed by introducing the dmo gene, encoding dicamba monooxygenase from *Stenotrophomonas maltophilia*, which catalyzes the oxidative demethylation of dicamba into the non-toxic compound 3,6-dichlorosalicylic acid (Behrens et al., 2007). These innovations allow farmers to apply 2,4-D or dicamba directly on soybean fields without injuring the crop, expanding weed control options. In practice, this technology was commercialized in cultivars known as Xtend^®^ soybeans, which combine tolerance to dicamba and glyphosate, providing growers with broader flexibility in herbicide use and playing an increasingly important role in weed management strategies in Brazil and worldwide.

The introduction of auxinic herbicide-tolerant soybean events has provided significant benefits in managing glyphosate-resistant and difficult-to-control broadleaf weeds, particularly in South and North America, where resistant populations of *Amaranthus* spp. have become one of the major challenges for soybean production. By enabling the use of multiple herbicide mechanisms of action, these technologies contribute to herbicide rotation strategies and delay the evolution of further resistance. However, auxinic herbicides are highly volatile and prone to drift, raising concerns about off-target damage to sensitive crops and natural vegetation. Consequently, stewardship measures such as buffer zones, application timing, and use of drift-reduction technologies are required to mitigate these risks (Egan et al., 2014).

As can be seen in Table 1, many soybean lines currently approved for commercial use, by CTNBio and other regulatory agencies, are stacked, which means that they combine more than one trait, either more than one herbicide tolerance or herbicide tolerance plus insect resistance, as a result of different genes introduced by genetic engineering. In the insect resistance category, GM soybean events mainly express resistance to insects from the Lepidopteran order. The genes of choice in this scenario come from the *cry* category. Cry proteins are specifically toxic to the insect orders Lepidoptera, Coleoptera, Hymenoptera and Diptera, and to nematodes. These proteins are produced by the *Bacillus thuringiensis* (Bt), a gram-positive pore-forming bacteria with entomopathogenic properties, being the reason why Bt is used as a microbial insecticide for improved resistance in plants by genetic modification, called Bt crops.

Briefly, Bt produces insecticidal proteins during the sporulation phase as parasporal crystals, which predominantly are comprised of one or more proteins, Cry and Cyt toxins, also called δ-endotoxins. These toxins, known as pore-forming toxins (PFT) are highly specific to their target insect, but are innocuous to humans, vertebrates, and plants, and are completely biodegradable (Bravo et al., 2007). The mode of action consists of these toxins’ interaction with specific receptors located on the host cell surface. In most cases, PFTs are activated by host proteases after receptor binding inducing the formation of an oligomeric structure that is insertion competent. Finally, membrane insertion is triggered, in most cases, by a decrease in pH that induces a molten globule state of the protein (Parker and Feil, 2005). Thus, in a few words, the primary action of Cry toxins is to lyse midgut epithelial cells in the target insect by forming pores in the apical microvilli membrane of the cells. These pores enable cations to flow into the cell, and water follows, possibly through aquaporins, causing the cells to swell and lyse. Minor damage might be healed by the insect, but major damage destroys the midgut epithelium, resulting in rapid cessation of feeding and eventual death of the insect.

Usually, Bt is used in commercial formulations in the form of sprays or liquid suspensions to be applied to the crop. Since the toxin is produced continuously for a longer time, it is comparatively better than chemical pesticides in terms of application and field management as well as cost and efficiency. In addition, the formulation solves some storage and stability problems. It not only stabilizes the biopesticide, but it also solves some other challenges such as handling the product, protecting it from environmental effects and enhancing the activities of agents like microbes in the field. The present-day’s formulation has additional benefits of speed killing, dry and wet (moisture), wind, rains, and characteristics of plants such as the chemistry of leaves.

In the context presented in this review and for commercial reasons, the idea of stacking different genes conferring diverse traits in a single variety was obvious. In general, stacked GMOs can be classified into four different types: 1) genes that confer resistance to multiple insect species; 2) the expression of the Bt insecticidal protein in parallel with tolerance to glyphosate herbicide; 3) genetic sequences ensuring a simultaneous tolerance to different types of herbicide; 4) other types of biotech traits capable of enhancing plant tolerance to drought and/or improving its nutritional content. 

Commercially, the adoption of stacked events was very positive. In 2011, stacked GMOs were cultivated in 42,2 Mhas -23.4% of the global area covered by transgenic seeds. Seven years later, in 2018, plantations with this technology reached 80,5 Mhas, representing 42% of the global area dedicated to GM crops (Seixas et al., 2022). In our country, the great jump in the adoption of stacked cultivars started in the 2013/2014 crop season with the release of Monsanto’s Soja Intacta™. This event expressed simultaneously both biotech traits, herbicide tolerance and insect resistance. In 5 years, Intacta™‘s cultivation area went from 2,3 Mhas in 2013 to 20,2 Mhas in 2018, making this cultivar the GMO with the largest diffusion during the 2010s (ISAAA, 2018).

The fast pace of dissemination of stacked GMOs in Brazil and worldwide has motivated a variety of studies about the economic and environmental impact of this technological improvement. As the main benefits of stacked seeds compared with single-trait biotech GMOs, these works point to the benefit in agricultural productivity, the farmers’ increasing profits, the decrease in the use of crop protection chemicals, and the reduction of carbon emissions (Waquil et al., 2013; Brookes, 2020). Besides that, in practical terms, stacked events deliver to growers, varieties with value-added as well as advantages and a range of crop management possibilities. The combination of herbicide-resistance traits for example allows the rotation of herbicides or the use of mixtures of herbicides that will greatly suppress several present or future herbicide-resistant weeds (Seixas et al., 2022).

However, there is one soybean event for drought tolerance approved by CTNBio in 2019 for commercial use named Verdeca HB4^®^ from Verdeca company, a joint venture between Arcadia Biosciences, Inc. (Nasdaq: RKDA) and Bioceres Crop Solutions Corp. (NYSE American: BIOX). This event was developed using *Agrobacterium tumefaciens* methodology and the gene inserted was *Hahb-4,* from *Helianthus annuus*. In addition to drought tolerance, HB4^®^ soybean also is glufosinate-tolerant, a broad-spectrum herbicide used to control a wide range of weeds, adding extra value to the event and a broader range of crop management possibilities to growers.

## Manuscripts related to the theme published from 2000’ to present

The systematic review using the Publish or Perish software and the terms transgenic OR genetic engineering OR genetically modified AND soybean AND Brazil resulted in 53 manuscripts up to date (2024). Most of them were produced between 2000 and 2009 (14) and 2010 and 2019 (23). Only four papers on the theme were published in the 90s and from 2020 to the present, 12 manuscripts addressed this topic. It’s important to highlight that most of the important traits to overcome agricultural issues such as pathogen and abiotic stress started to be research’s focus by the middle to end of the 2000s, with most of the papers on these themes being produced in the 2000’s decade (Table 2). 

**Table 2-:** List of manuscripts published in Brazil on the topic of genetic engineering on soybean from the 1990s to the present. Authors, year, title of the manuscript, the characteristics or traits related and the link to access the paper are presented.

Authors	Year	Title	Characteristic/Trait	Article/URL
Rech *et al.*	1988	Agrobacterium rhizogenes Mediated Transformation of the Wild soybeans *Glycine canescens* and *G. clandestine*: Production of transgenic Plants of G. canescens	Method development	https://academic.oup.com/jxb/article-abstract/39/9/1275/552039
Rech *et al.*	1989	Expression of a chimaeric kanamycin resistance gene introduced into the wild soybean *Glycine canescens* using a cointegrate Ri plasmid vector	Method development	https://link.springer.com/article/10.1007/BF00735773
Tulmann Neto; Peixoto	1990	Early maturing and good yield mutants in soybean (*Glycine max* (L.) Merr.) in Brazil.	Early maturing and yield	https://www.cabdirect.org/cabdirect/abstract/19911620593
Santarém; Finer	1999	Transformation of soybean [*Glycine max* (L.) Merrill] using proliferative embryogenic tissue maintained on semi-solid medium	Method development	https://link.springer.com/article/10.1007/s11627-999-0067-0
Droste *et al.*	2002	Transgenic fertile plants of soybean [*Glycine max* (L.) Merrill] obtained from bombarded embryogenic tissue	Method development	https://link.springer.com/article/10.1023/A:1020370913140
Contim *et al.*	2003	The soybean sucrose binding protein gene family: genomic organization, gene copy number and tissue-specific expression of the SBP2 promoter	Sucrose uptake	https://academic.oup.com/jxb/article/54/393/2643/503895
Borém *et al.*	2003	A plant binary vector with an antisense soybean UDP-glucose dehydrogenase gene	Pathogen resistance	https://pdfs.semanticscholar.org/d489/e1b73a13822a6c2ae7523dd0561b1f9972e3.pdf
Sachet	2005	Avaliação da eficiência de co-transformação de soja (*Glycine max* (L.) Merrill) via bombardeamento de partículas utilizando um gene de seleção e um gene de …	Method development	https://www.lume.ufrgs.br/handle/10183/7358
Nunes *et al.*	2006	RNAi-mediated silencing of the myo-inositol-1-phosphate synthase gene (GmMIPS1) in transgenic soybean inhibited seed development and reduced phytate content	RNAi	https://link.springer.com/article/10.1007/s00425-005-0201-0
Robić *et al.*	2006	Downstream process engineering evaluation of transgenic soybean seeds as host for recombinant protein production	Method development	https://www.sciencedirect.com/science/article/pii/S1369703X06002154
Tinoco *et al.*	2006	Radiation as a tool to remove selective marker genes from transgenic soybean plants		https://link.springer.com/article/10.1007/s10535-005-0091-9
Dang and Wei	2007	An optimized Agrobacterium-mediated transformation for soybean for expression of binary insect resistance genes	Insect resistance	https://doi.org/10.1016/j.plantsci.2007.06.010
Homrich	2008	Plantas transgênicas de soja (*Glycine max* (L.) Merril) resistentes à lagarta *Anticarsia gemmatalis*	Insect resistance	https://www.lume.ufrgs.br/handle/10183/13612
Homrich *et al.*	2008	Resistance to *Anticarsia gemmatali*s Hübner (Lepidoptera, Noctuidae) in transgenic soybean (*Glycine max* (L.) Merrill Fabales, Fabaceae) cultivar IAS5 expressing a …	Insect resistance	https://doi.org/10.1590/S1415-47572008000300020
Fernandes	2009	Caracterização molecular e biológica de begomovírus de soja (*Glycine ma*x) e leiteiro (*Euphorbia heterophylla*) e resistência a vírus mediada por RNA interferente …	RNAi	https://www.locus.ufv.br/handle/123456789/1023
Hernandez-Garcia *et al.*	2009	A soybean (*Glycine max*) polyubiquitin promoter gives strong constitutive expression in transgenic soybean	Method development	https://link.springer.com/article/10.1007/s00299-009-0681-7
Marra *et al.*	2009	Protective effects of a cysteine proteinase propeptide expressed in transgenic soybean roots		https://www.sciencedirect.com/science/article/pii/S0196978109000400
Valente *et al.*	2009	The ER luminal binding protein (BiP) mediates an increase in drought tolerance in soybean and delays drought-induced leaf senescence in soybean and tobacco	Drought tolerance	https://academic.oup.com/jxb/article-abstract/60/2/533/630885
Cunha *et al.*	2010	High resistance to Sclerotinia sclerotiorum in transgenic soybean plants transformed to express an oxalate decarboxylase gene		https://bsppjournals.onlinelibrary.wiley.com/doi/abs/10.1111/j.1365-3059.2010.02279.x
Robić *et al.*	2010	Transgenic soybean seed as protein expression system: Aqueous extraction of recombinant β-glucuronidase		https://link.springer.com/article/10.1007/s12010-009-8637-5
Cunha *et al.*	2011	Accumulation of functional recombinant human coagulation factor IX in transgenic soybean seeds		https://link.springer.com/article/10.1007/s11248-010-9461-y
Cunha *et al.*	2011	Expression of functional recombinant human growth hormone in transgenic soybean seeds		https://link.springer.com/article/10.1007/s11248-010-9460-z
Polizel *et al.*	2011	Molecular, anatomical and physiological properties of a genetically modified soybean line transformed with rd29A: AtDREB1A for the improvement of drought …	Drought tolerance	https://www.alice.cnptia.embrapa.br/handle/doc/916042
Reis *et al.*	2011	The binding protein BiP attenuates stress-induced cell death in soybean via modulation of the N-rich protein-mediated signaling pathway		https://academic.oup.com/plphys/article-abstract/157/4/1853/6109196
Wiebke-Strohm *et al.*	2011	Transgenic fertile soybean plants derived from somatic embryos transformed via the combined DNA-free particle bombardment and Agrobacterium system	Method development	https://link.springer.com/article/10.1007/s10681-010-0249-1
Wiebke-Strohm *et al.*	2012	Ubiquitous urease affects soybean susceptibility to fungi		https://link.springer.com/article/10.1007/s11103-012-9894-1
Barbosa *et al.*	2013	Overexpression of the ABA-dependent AREB1 transcription factor from Arabidopsis thaliana improves soybean tolerance to water deficit	Drought tolerance	https://link.springer.com/article/10.1007/s11105-012-0541-4
Engels *et al.*	2013	Introduction of the rd29A: AtDREB2A CA gene into soybean (*Glycine max* L. Merril) and its molecular characterization in leaves and roots during dehydration	Drought tolerance	https://www.scielo.br/pdf/gmb/v36n4/v36n4a15.pdf
Cunha *et al.*	2014	Recombinant biosynthesis of functional human growth hormone and coagulation factor IX in transgenic soybean seeds		https://link.springer.com/article/10.1186/1753-6561-8-S4-P112
Leite *et al.*	2014	Overexpression of the activated form of the AtAREB1 gene (AtAREB1^ QT) improves soybean responses to water deficit.	Drought tolerance	https://www.alice.cnptia.embrapa.br/handle/doc/993429
Murad *et al.*	2014	Expression, purification and analysis of the anti-HIV Cyanovirin-N produced in transgenic soybeans seeds		https://bmcproc.biomedcentral.com/articles/10.1186/1753-6561-8-S4-P105
Rolla *et al.*	2014	Phenotyping soybean plants transformed with rd29A: AtDREB1A for drought tolerance in the greenhouse and field	Drought tolerance	https://link.springer.com/article/10.1007/s11248-013-9723-6
Weber *et al.*	2014	Expression of an osmotin-like protein from Solanum nigrumconfers drought tolerance in transgenic soybean	Drought tolerance	https://link.springer.com/article/10.1186/s12870-014-0343-y
Honna *et al.*	2016	Molecular, physiological, and agronomical characterization, in greenhouse and in field conditions, of soybean plants genetically modified with AtGolS2 gene for …	Drought tolerance	https://link.springer.com/article/10.1007/s11032-016-0570-z
Kawashima *et al.*	2016	A pigeonpea gene confers resistance to Asian soybean rust in soybean		https://www.nature.com/articles/nbt.3554
Marinho *et al.*	2016	Characterization of molecular and physiological responses under water deficit of genetically modified soybean plants overexpressing the AtAREB1 transcription factor	Drought tolerance	https://link.springer.com/article/10.1007/s11105-015-0928-0
Pimenta *et al.*	2016	The stress-induced soybean NAC transcription factor GmNAC81 plays a positive role in developmentally programmed leaf senescence	Drought tolerance	https://academic.oup.com/pcp/article-abstract/57/5/1098/2223406
Conforte *et al.*	2017	Isolation and characterization of a promoter responsive to salt, osmotic and dehydration stresses in soybean	Salt, osmotic and dehydration tolerances	https://doi.org/10.1590/1678-4685-GMB-2016-0052
Fuganti-Pagliarini *et al.*	2017	Characterization of soybean genetically modified for drought tolerance in field conditions	Drought tolerance	https://www.frontiersin.org/articles/10.3389/fpls.2017.00448/full
Soto *et al.*	2017	Efficient particle bombardment-mediated transformation of Cuban soybean (INCASoy-36) using glyphosate as a selective agent	Method development	https://link.springer.com/article/10.1007/s11240-016-1099-x
Rechenmacher *et al.*	2019	Endogenous soybean peptide overexpression: An alternative to protect plants against root-knot nematodes	Biotic stress - Nematodes	https://www.sciencedirect.com/science/article/pii/S2452072119301121
Fuganti-Pagliarini *et al.*	2020	Drought-tolerant soybean development: evaluation of GM lines under greenhouse and field conditions.	Drought tolerance	https://www.alice.cnptia.embrapa.br/handle/doc/1128419
Molinari *et al.*	2020	Overexpression of AtNCED3 gene improved drought tolerance in soybean in greenhouse and field conditions	Drought tolerance	https://doi.org/10.1590/1678-4685-GMB-2019-0292
Araujo *et al.*	2021	Overexpression of DUF538 from Wild Arachis Enhances Plant Resistance to Meloidogyne spp.	Nematode resistance	https://www.mdpi.com/1035204
Berchembrock *et al.*	2021	Suppression of ERECTA Signaling Impacts Agronomic Performance of soybean (*Glycine max* (L) Merril) in the Greenhouse		https://www.frontiersin.org/articles/10.3389/fpls.2021.667825/full
Pérez *et al.*	2021	Host‐induced gene silencing for engineering resistance to Fusarium in soybean		https://bsppjournals.onlinelibrary.wiley.com/doi/abs/10.1111/ppa.13299
Rodrigues *et al.*	2021	BiP-overexpressing soybean plants display accelerated hypersensitivity response (HR) affecting the SA-dependent sphingolipid and flavonoid pathways		https://www.sciencedirect.com/science/article/pii/S0031942221000510
Koltun *et al.*	2022	Uncovering the roles of hemoglobins in soybean facing water stress	Drought tolerance	https://www.sciencedirect.com/science/article/pii/S0378111921006508
Marinho *et al.*	2022	Overexpression of full-length and partial DREB2A enhances soybean drought tolerance	Drought tolerance	https://mecenaspublishing.com/journals/index.php/asbjournal/article/view/141
Basso *et al.*	2022	Overexpression of a soybean Globin (GmGlb1-1) gene reduces plant susceptibility to *Meloidogyne incognita*	Nematode resistance	https://link.springer.com/article/10.1007/s00425-022-03992-2
Fragoso *et al.*	2022	Functional characterization of the pUceS8.3 promoter and its potential use for ectopic gene overexpression		https://link.springer.com/article/10.1007/s00425-022-03980-6
Rodrigues *et al.*	2023	BiP-overexpressing soybean shows differential hypersensitivity response (HR) altering protein and metabolite profiles involved in the plant defense		https://link.springer.com/article/10.1007/s40502-023-00717-9
Basso *et al.*	2023	Overexpression of the GmEXPA1 gene reduces plant susceptibility to *Meloidogyne incognita*	Nematode resistance	https://link.springer.com/article/10.1007/s00299-022-02941-3
de Oliveira Bosatto *et al.*	2024	The Overexpression of *Solanum nigrum* Osmotin (SnOLP) Boosts Drought Response Pathways in Soybean	Drought tolerance	https://link.springer.com/article/10.1007/s11105-024-01452-7

The systematic review applying the terms transgenic OR genetic engineering OR genetically modified AND soybean AND all other 11 South American countries (Argentina, Bolivia, Chile, Colombia, Ecuador, Guyana, Paraguay, Peru, Suriname, Uruguay, and Venezuela) did not return any manuscript published on basic and applied research on genetically modified soybean. Despite that, as already highlighted in this text, some of these South American countries, such as Argentina, Colombia, Paraguay, and Uruguay grow GM soybean varieties developed by other countries, being a “consumer” of GM technology (Table 1). 

## Future perspectives of soybean genetic engineering in Brazil

Although soybean production in Brazil has reached numbers that put our country as the second and for some crop seasons, as the first world producer, the challenges in the field never end, demanding constant research focused on maintaining production while facing current and upcoming abiotic and biotic adverse environmental challenges. 

Plant biotechnology has done a good job so far in developing single and stacked GMOs, to fulfill present demands, however, the stacking of many traits tends to compromise the myriad of other agronomic characteristics not controlled by the transferred genes, which can ultimately reduce the physiological quality and productivity of soybean (Seixas et al., 2022). But ahead, there’s hope. There is an expectation that the new editing technologies such as the CRISPR-Cas9 system and variants will revolutionize the development process of agronomic traits, enabling the expression of a much larger number of traits than the ones currently observed in the GMOs existent in the market (Vats et al., 2019), much probably without compromising other important traits, due to its precision feature. Besides that, these new tools are here to stay in agriculture for many other reasons: value added to commodities and consumer’s crops; technology tool-boxes are more specific and relatively simpler to apply, avoiding exiting scientific bias of other methodologies; these crops can also overcome regulation restrictions and costs, being accessible to a greater number of research institutions, which brings “new players” to the field of development of products, increasing competitiveness and choices to the population; and finally outcomes products can be considered non-GM, breaking taboos of producers and especially from the final consumers (Fernandes et al., 2022). 

The tendency for the future is to have more and more products developed with these technologies on the field and market shelves. It is a path of no return and the secret to the technology’s success will be reliable regulation, labelling, and how the message of these editing crops and related products will be delivered to consumers (Fernandes et al., 2022). For soybean, this is already happening. By September 2022, Brazil had three soybean events obtained through the New Breeding Technologies and approved by CTNBio for commercial use; two from GDM Genetica do Brasil S.A.; and one from Embrapa Soybean. 

The events from GDM Genetica do Brasil S.A. show lower levels of raffinose and drought tolerance. Raffinose is a soluble sugar present in soybeans, known as an anti-nutritional factor. Thus, when ingested from feed made with soybean meal, it causes gas, intestinal discomfort, and decreases feed energy and weight gain in monogastric animals, such as birds, and swine. The edited event aimed to lower levels of raffinose. According to the researchers who developed the event, the new version of the protein of this gene resulted in a reduction of 75% of raffinose and 50% of stachyose in the seeds, creating value for the soy business chain. The edited line use in poultry and swine farming should increase nutritional quality, allowing producers to spend less on fattening and allowing faster weight gain in animals (Surian, 2022).

Due to its importance in the feed industry, managing anti-nutritional factors was also the target of Embrapa Soybean. The recently approved edited line has an anti-nutritional factor deactivated, a lectin, and was obtained using CRISPR technology. As described these factors make the soy grain difficult to digest in monogastric animals, which have a stomach with reduced storage capacity. Thus, the use of soybeans for these ends depends on thermal processing to inactivate these anti-nutritional factors, increasing production costs. This edited event can potentially enable the reduction of costs in the use of soy for animal feed, guaranteeing the nutritional quality of the grain. In addition, this technology can now be applied to other varieties adapted to different production regions opening Embrapa’s portfolio. Besides that, it is important to highlight that as being considered non-GMO, the commercial release of this event is faster, reducing costs and facilitating the entry of products on the market with assured biosecurity. The commercial release of this event places Embrapa, the first public company to have an approved edited soybean line, in the same research and commercial levels as big biotech companies.

In conclusion, and given all the content presented here in this review, soybean research in Brazil at first provided the planting of soybeans in regions not previously explored for this purpose; later, the use of single and stacked GM events solved agricultural issues, promoted better crop management, trying to keep productivity and mitigate losses and, in the next future, through editing tools, soybean Brazilian research will continue to provide solutions and improvements to national agriculture, to feed industry as well as human consumption.

## Data Availability

This manuscript is a review article and does not include any new data generated or analyzed by the authors. All data discussed are available in the published literature cited throughout the text.
